# Fluorescent Probes for Aldehyde Detection in Biological Systems: Design Principles, Spectroscopy, and Emerging Applications

**DOI:** 10.3390/s26165108

**Published:** 2026-08-12

**Authors:** Eva-Maria Bryan, Ozlem Dilek

**Affiliations:** Department of Chemistry and Biochemistry, College of Science, Institute for Advanced Biomedical Research, George Mason University, Manassas, VA 20110, USA; erudler@gmu.edu

**Keywords:** aldehydes, fluorescent probes, formaldehyde, malondialdehyde, 4-hydroxynonenal, acrolein, reaction-based sensing, near-infrared, two-photon excitation, FLIM, ratiometric imaging, ferroptosis, bioimaging, oxidative stress, one-carbon metabolism

## Abstract

Reactive aldehydes—formaldehyde (FA), malondialdehyde (MDA), 4-hydroxynonenal (4-HNE), and acrolein—occupy a central role in epigenetic regulation, lipid peroxidation, ferroptosis, and cardiovascular disease, yet their transient nature and low intracellular concentrations have long made them difficult to quantify in living systems. Over the past decade, reaction-based small-molecule fluorescent probes have emerged as the principal tool for addressing this challenge, and this review provides a systematic account of that progress. We compare the six main conjugation chemistries that underlie current probe design—2-aza-Cope/Mannich cascade, hydrazone/oxime formation, Michael addition, Schiff base condensation, and 2-aminothiophenol cyclization—alongside the three photophysical strategies used to convert these reactions into quantitative signals: intensity turn-on, ratiometric dual-emission, and fluorescence lifetime imaging (FLIM). Attention is given to advances reported between 2020 and 2025, including organelle-targeted ratiometric formaldehyde sensors (MitoRFAP-2, NucRFAP-2), the first ratiometric acrolein probe for visualizing ferroptosis, lysosome-targeted malondialdehyde reporters for atherosclerosis staging, and near-infrared-compatible platforms validated in three-dimensional organoids and in vivo models. This review also critically examines the limitations that continue to constrain the field, including insufficient selectivity testing under physiologically relevant conditions, the pH-dependence of hydrazone equilibria, the frequently overlooked distinction between probes that report free aldehyde concentration and those that report ALDH enzyme activity, and the continued absence of reversible, real-time sensors. Closing these gaps will determine whether aldehyde imaging grows from a set of smart probes into a quantitative, widely trusted platform for studying redox and carbonyl biology in living systems. Reaching that point will depend on three criteria: better NIR fluorophores, effective bioconjugation chemistry, and machine-learning tools that guide probe design.

## 1. Introduction

Aldehydes have a central position in cell biology. They are not simply metabolic waste products awaiting clearance; in several of the most consequential pathways in mammalian physiology, the aldehyde itself is the signal, the damage, or both. Formaldehyde offers perhaps the clearest illustration of this principle. Generated as a stoichiometric byproduct of histone demethylation by LSD1/KDM1A, its production is mechanistically coupled to chromatin remodeling, such that the concentration and subcellular distribution of formaldehyde at any given moment is itself a readout of demethylase activity [1,2]. The implications extend well beyond this single enzymatic context: formaldehyde now appears to function as a one-carbon signaling molecule that bridges one-carbon metabolism, epigenetic methylation, and nuclear regulation [1,2,3,4,5], placing a molecule once regarded purely as a toxicant at the center of a regulatory network central to genome function.

A parallel logic can be derived for the lipid-derived aldehydes. Acrolein and 4-hydroxynonenal (4-HNE) arise as the terminal electrophilic products of lipid peroxidation, generated during inflammatory signaling and during the execution of ferroptotic cell death [6,7,8]. Acrolein carries an additional environmental dimension, accumulating from tobacco smoke exposure and forming protein adducts whose mechanistic role in Parkinson’s disease and ferroptosis-associated neurodegeneration is now well established [6,8]. Malondialdehyde (MDA), meanwhile, accumulates wherever oxidative stress is sustained, reacting with lysine residues, guanine bases, and membrane phospholipids to generate the adduct signature now recognized as a defining molecular feature of atherosclerosis, Alzheimer’s disease, and renal injury [9,10,11,12]. Across these examples, a single theme recurs: the biological consequence of aldehyde chemistry is inseparable from where and when that chemistry takes place.

This is precisely the information that conventional analytical methods cannot supply. DNPH derivatization, GC-MS, and HPLC remain the established gold standards for aldehyde quantification and offer excellent sensitivity but are known as destructive method since each reports a bulk concentration averaged across a homogenized sample, discarding spatial information entirely and requiring conditions incompatible with measurement in living cells [13,14,15]. Imaging these aldehydes directly in living systems, with spatial, temporal, and subcellular resolution, closes precisely this gap, transforming a transient chemical event into a measurement that can be localized to a specific organelle, cell, or tissue region at a defined point in time. Reaction-based fluorescent probes have emerged as the principal tool for achieving this: by design, they generate a specific optical signal only at the time and place where the target aldehyde chemistry happens, converting reactive into spectroscopic data that can be resolved spatially and followed over time.

**Figure 1:**
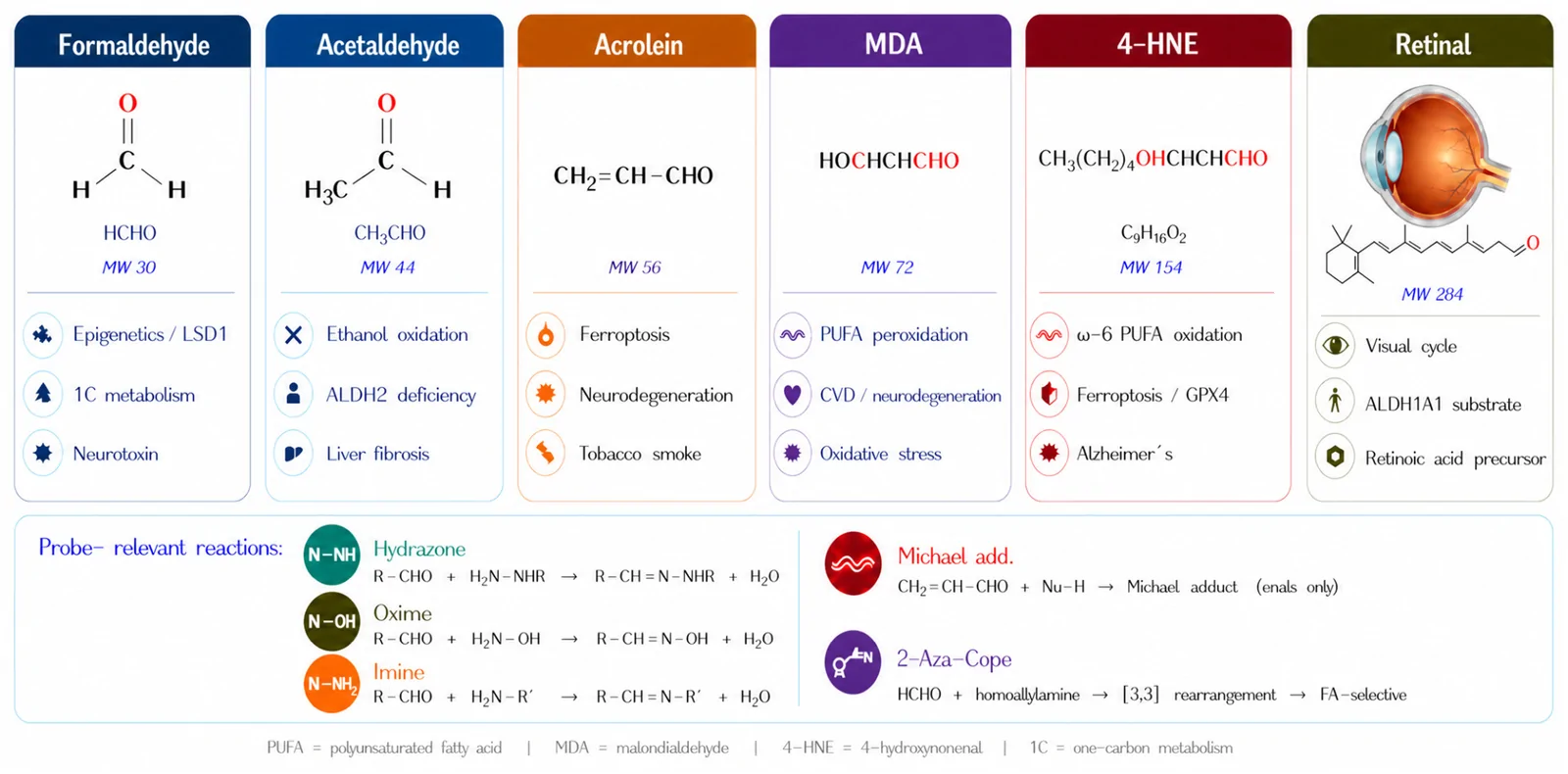
Biologically relevant aldehydes and key probe-relevant reactions. (**Top**): the six probe-targeted aldehyde species with 3D molecular structures, formulae, molecular weights, and primary pathological roles. (**Bottom**): The five detection reaction strategies used to detect them (hydrazone, oxime, imine, Michael addition, 2-aza-Cope). Abbreviations: PUFA, polyunsaturated fatty acid; MDA, malondialdehyde; 4-HNE, 4-hydroxynonenal; 1C, one-carbon metabolism. References: [1,6,7,9,10,19].

This review examines that chemistry comprehensively addressing probe design principles, the photophysical strategies used to convert a chemical reaction into a quantitative optical readout, and the biological applications these probes have enabled, with particular emphasis on advances published between 2020 and 2025. One distinction is maintained throughout and, in our view, deserves explicit attention at the outset, because its neglect has propagated a recurring source of misinterpretation in this literature: probes that report free aldehyde concentration and probes that report aldehyde dehydrogenase (ALDH) enzyme activity measure fundamentally different biological quantities. The two are not interchangeable and treating them as such has led to demonstrably incorrect biological conclusions in published work [13,16]. We return to this distinction at each point in the review where it bears on the correct interpretation of experimental results. Figure 1 summarizes the six aldehyde species addressed in this review alongside the five probe-relevant reaction strategies that structure the discussion in Section 2.

Two recent surveys inform the scope of the present review. Wills et al. (2024) introduced the tunable 2-aminothiophenol platform for total-aldehyde sensing discussed in Section 2.6, and Semwal and Das (2025) surveyed selective aldehyde chemosensors from a primarily chemistry-of-detection standpoint [17,18]. The present review is distinguished on four axes. First, it is organized around the practical decision a researcher faces, namely which conjugation chemistry and which photophysical readout fit a given biological question, rather than around fluorophore families alone. Second, it treats the readout strategy (turn-on, ratiometric, FLIM, two-photon) as a design axis of equal weight to the recognition chemistry and makes explicit the interpretive limits of each (Section 3). Third, it foregrounds a distinction that is frequently conflated in the primary literature yet rarely stated in prior reviews: probes that report free aldehyde concentration versus those that report ALDH enzyme activity (Section 4.4). Fourth, it concentrates on 2020–2025 advances (organelle-targeted ratiometric formaldehyde sensors, the first ratiometric acrolein probe, and lysosomal MDA reporters for disease staging) and closes with concrete, testable directions (NIR-II fluorophores, reversible oxime sensing, and computational design) grounded in adjacent, already validated fields.

### 1.1. Aldehydes: Chemical Reactivity and Biological Origins

The aldehydic carbonyl is highly electrophilic, particularly in formaldehyde where no alkyl substituent provides steric shielding. The biologically relevant aldehydes span from formaldehyde (MW 30) to retinal (MW 284), and their differential reactivity, hydration equilibria, and subcellular localizations collectively determine what probe chemistry can detect them reliably [7,19].

The present review concentrates on the four aldehydes whose probe development has been most active (formaldehyde, malondialdehyde, 4-hydroxynonenal, and acrolein) together with acetaldehyde and retinal, which are included in Figure 1 for chemical and biological context (acetaldehyde as the ALDH2 substrate central to the ALDH2*2 polymorphism, and retinal as the ALDH1A1-processed visual-cycle and retinoic-acid precursor). Several further aldehydes are biologically important and, although outside the primary scope here, merit brief mention. Glyoxal and methylglyoxal are reactive dicarbonyls that drive advanced-glycation-end-product formation in diabetes and hyperglycemia and are typically detected by o-phenylenediamine-type condensation rather than the mono-aldehyde chemistries emphasized here; methylglyoxal in particular is a standard cross-reactivity control in enal-probe selectivity panels (Section 2.4). Longer-chain lipid-peroxidation aldehydes such as trans-2-hexenal and crotonaldehyde share the α,β-unsaturated Michael-acceptor motif exploited for acrolein and 4-HNE and are likewise used as selectivity controls. These species are noted where they bear on selectivity, while the detailed probe-by-probe treatment remains focused on the four highest-activity targets.

#### 1.1.1. Nucleophilic Addition and Protein Carbonylation

Lysine ε-amines, histidine imidazoles, guanine, and adenine all react with aldehydes to form Schiff base adducts that can progress to Amadori products or covalent crosslinks [7,19]. Cysteine and glutathione react through the sulfur nucleophile, forming thioether Michael adducts that are the primary cellular detoxification route for α,β-unsaturated aldehydes. Collectively termed protein carbonylation, these modifications range from potentially signaling-relevant events to genotoxic crosslinking when reactive aldehydes overwhelm clearance capacity [1,14]. We have contributed directly to this area through development of bioorthogonal fluorescent probes for labeling carbonylated proteins in living cells, enabling real-time imaging of protein carbonylation dynamics under oxidative challenge [14,20,21].

#### 1.1.2. Enzymatic Metabolism: The ALDH Superfamily

The aldehyde dehydrogenase (ALDH) superfamily of 19 human isozymes converts aldehydes to carboxylic acids using NAD^+^/NADP^+^ [19,22]. ALDH2 in the mitochondrial matrix clears acetaldehyde and lipid-derived aldehydes; the ALDH2*2 loss-of-function polymorphism (~560 million carriers) dramatically impairs this clearance [22]. Subcellular-targeted ratiometric probes have directly quantified how ALDH2 activity governs steady-state mitochondrial FA pools, which is a result inaccessible to non-targeted probes [3]. The interplay between aldehyde production, ALDH-mediated clearance, and GSH conjugation determines the free aldehyde concentration available to any probe, a critical parameter addressed in Section 4.4.

#### 1.1.3. Hydration Equilibria and pH Dependence

Small aldehydes exist in pH-dependent equilibrium with gem-diol hydrates. Formaldehyde is ~99.9% hydrated at physiological pH; the free aldehyde pool is continuously replenished as it is consumed [23,24]. Hydrazone-forming probes compete kinetically with water for this free fraction, and this competition is pH-dependent. A probe calibrated at cytoplasmic pH 7.4 may respond very differently in lysosomes at pH 4.5 [23,24]—a practical source of non-reproducible selectivity data addressed in Section 5.1.

### 1.2. Why Are Reactive Aldehydes Difficult to Study in Living Systems?

Three properties make reactive aldehydes challenging live-cell imaging targets: short effective half-lives (sub-minute for FA; transient for lipid-derived species), structural similarity between aldehyde classes that defeats non-selective probe chemistries, and low basal intracellular concentrations (low-µM to nM) demanding exceptional signal-to-background ratios [2,16,20]. Additionally, 4-HNE and acrolein rapidly conjugate with cytoplasmic GSH (~5 mM) and protein thiols, depleting the free fraction available to probe nucleophiles—making GSH inclusion in selectivity panels a non-negotiable experimental standard [6,7].

### 1.3. The Case for Fluorescence-Based Detection

**Figure 2:**
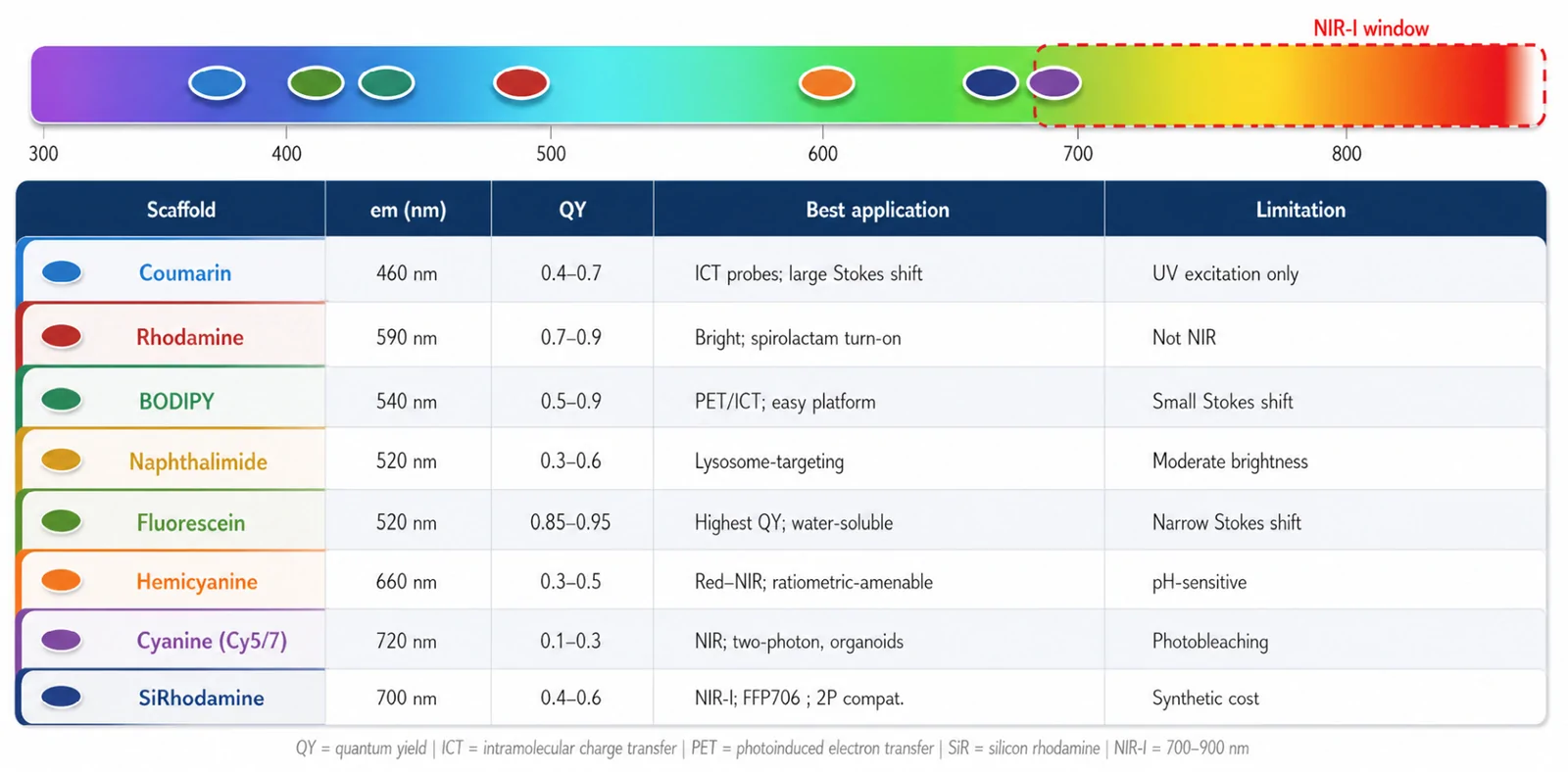
Fluorophore core scaffolds for aldehyde-responsive probes, arranged by emission wavelength. The NIR-I window (700–900 nm) is highlighted. For each scaffold: emission wavelength (nm), quantum yield (QY) range, best application, and primary limitation. References: [26,27,29,30].

Fluorescence imaging is non-destructive, compatible with repeated live-specimen measurements, and highly sensitive across a spectral range from UV through NIR-II (1000–1700 nm) [25,26,27,28]. The NIR-I window (700–900 nm) minimizes hemoglobin and melanin absorption while suppressing endogenous chromophore autofluorescence—the practical target for probes intended for live-tissue imaging. Ratiometric dual-emission and FLIM readouts, discussed in Section 3, provide concentration-independent quantitation essential for disease-model applications. Figure 2 illustrates the fluorophore core scaffolds spanning this emission range and their associated quantum yields.

## 2. Chemical Design Strategies for Aldehyde-Responsive Probes

**Figure 3:**
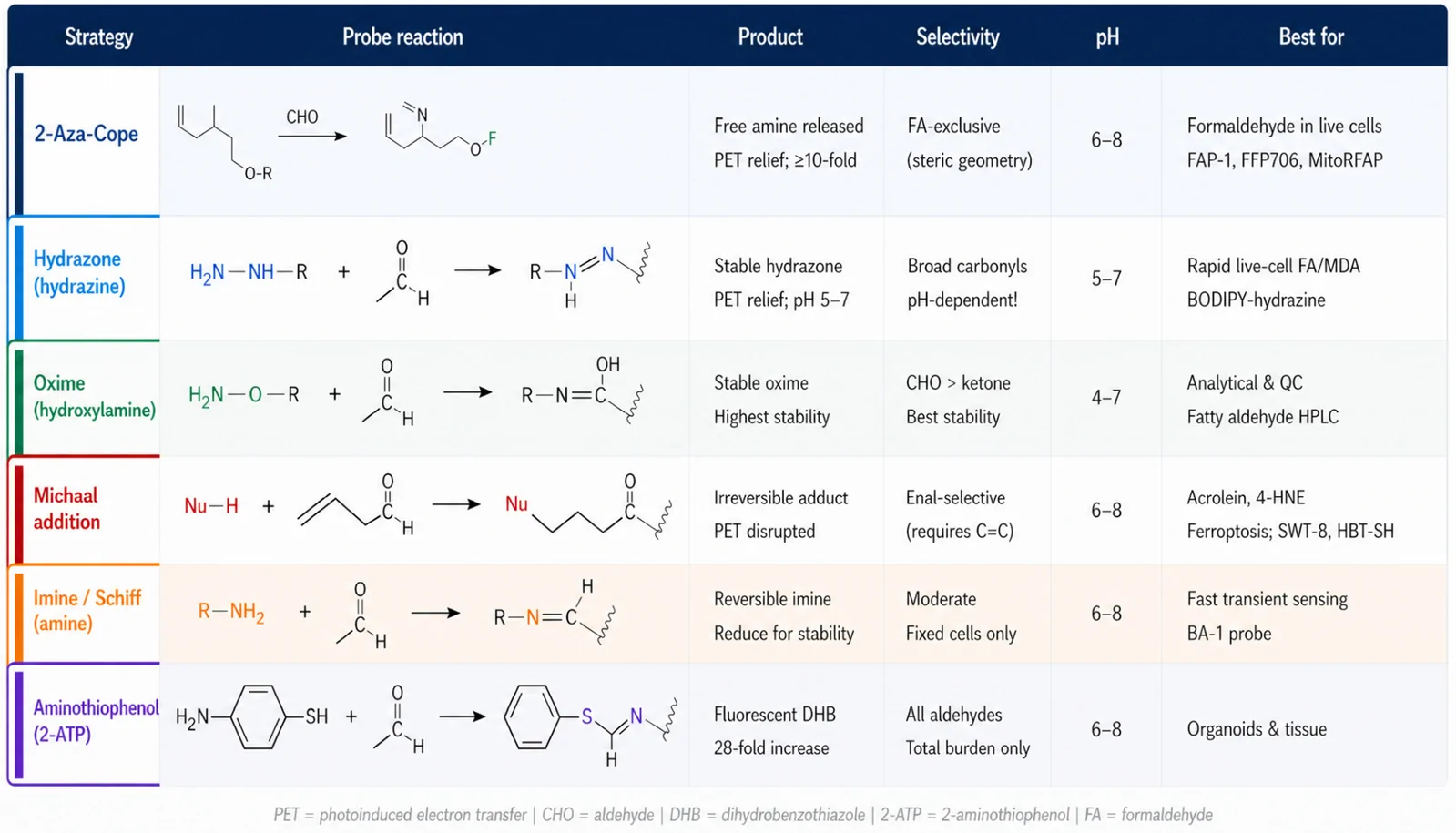
Comparative overview of the six aldehyde probe conjugation strategies: mechanism, product, selectivity profile, pH operating range, and optimal biological application. References: [1,2,3,4,6,8,15,17,23,24,31,32,33].

The choice of probe chemistry determines what an aldehyde sensor can detect, how quickly it responds, and under what conditions it provides reliable data. In the intracellular environment, a probe trigger must react preferentially with the target aldehyde over structurally similar species, competing ketones, and millimolar GSH—all at rates sufficient to capture a transient signal. Six chemical strategies have been established, each with distinct trade-offs. Figure 3 presents a comparative overview and Table 1 provides numerical parameters.

**Table 1:** Comparative summary of aldehyde probe conjugation strategies. DHB = dihydrobenzothiazole; FA = formaldehyde; QC = quality control; 1C = one-carbon metabolism. References: [1,2,3,4,6,8,15,17,23,24,31,32,33,34].

Probe Type	Product	Rate	Reversibility	pH Range	Selectivity	Optimal Use	Key Refs
**Hydrazine**	Hydrazone	Fast	Moderate (pH-dep.)	5–7	Broad carbonyls	Rapid live-cell FA/MDA	[31,32]
**Hydrazide**	Acyl hydrazone	Moderate	Low	5–7	Improved	In vivo tissue imaging	[34]
**Hydroxylamine**	Oxime	Moderate	Very low	4–7	CHO > ketone	Analytical/QC	[15]
**2-Aza-Cope**	Amine released	Moderate	Irreversible	6–8	FA-exclusive	FA/epigenetics/1C	[1,2,3,4]
**Michael addition**	Michael adduct	Fast	Irreversible	6–8	Enal-selective	Acrolein/4-HNE/ferroptosis	[6,8]
**Amine/Schiff**	Imine	Very fast	Reversible	6–8	Moderate	Fast transient sensing	[33]
**Aminothiophenol**	DHB adduct	Moderate	Low–moderate	6–8	All aldehydes	Total burden; organoids	[17]

### 2.1. The 2-Aza-Cope/Mannich Cascade: Formaldehyde Selectivity by Mechanistic Design

The 2-aza-Cope/Mannich cascade developed by Brewer and Chang [1] achieves selectivity through reaction mechanism rather than probe structure tuning. Homoallylamine triggers condense with FA to form an iminium ion that undergoes a [3,3]-sigmatropic rearrangement through a chair-like six-atom transition state, followed by hydrolysis to release the fluorophore amine and activate fluorescence [1]. Selectivity for FA is mechanistic: formaldehyde bears no α-substituents and populates the chair-like TS without steric penalty, whereas acetaldehyde, propanal, and larger aldehydes introduce steric clash that prevents productive rearrangement—selectivity intrinsic to geometry, not to probe electronics.

**Figure 4:**
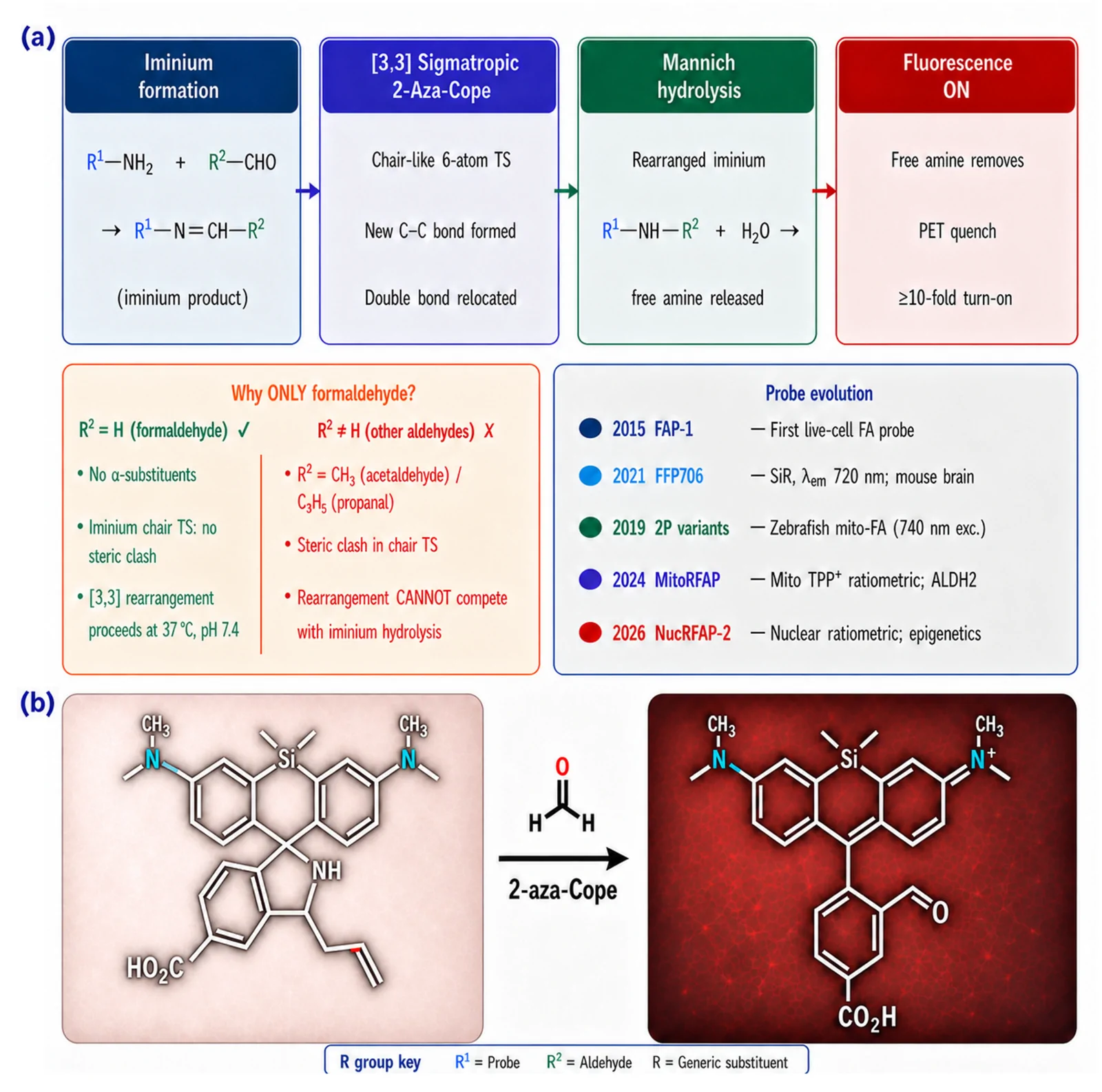
(**a**) 2-Aza-Cope/Mannich cascade—formaldehyde-selective mechanism and probe evolution timeline. Top: four-step reaction flow (iminium formation → [3,3] sigmatropic rearrangement → Mannich hydrolysis → fluorescence ON). Bottom left: mechanistic basis of FA selectivity—HCHO has no α-substituents (chair-TS accessible); other aldehydes face steric clash (rearrangement cannot compete with iminium hydrolysis). Bottom right: probe evolution from FAP-1 (2015 [1]) through FFP706 (2021 [2]), 2P variants (2019 [36]), MitoRFAP (2024 [3]), and NucRFAP-2 (2026 [4]). (**b**) Chemical structures of the silicon rhodamine homoallylamine probe FAP-1 (left, probe; right, HCHO adduct after cascade) overlaid on red-channel confocal cell images, showing NIR emission (λem ≈ 720 nm) compatible with tissue imaging. Chemical structures in panel (**b**) are reproduced directly from Brewer and Chang, J. Am. Chem. Soc. 2015 [1]. Abbreviations: TS, transition state; FA, formaldehyde; PET, photoinduced electron transfer; SiR, silicon rhodamine; TPP^+^, triphenylphosphonium.

Du and colleagues performed systematic SAR of the homoallylamine trigger, demonstrating N-p-methoxybenzyl substitution stabilizes the probe while accelerating iminium formation, compatible with the silicon rhodamine scaffold (FFP706, λex/λem ≈ 650/720 nm) [2]. Two-photon-excitable variants [35,36] enabled the first visualization of basal mitochondrial FA in live zebrafish larvae [36]. The most significant recent extensions are the organelle-targeted ratiometric probes from the Chang laboratory: MitoRFAP-2 (mitochondria, TPP^+^ targeting, 2024 [3]) and NucRFAP-2 (nucleus, NLS targeting, 2026 [4]), which provided the first quantitative comparison of organelle-specific FA pools. An ER-targeting two-photon FA probe [37] enabled in situ monitoring of formaldehyde-induced ER stress in neuroinflammation models. All 2-aza-Cope probes integrate cumulative FA exposure rather than reporting instantaneous concentration (Section 5.3). Figure 4 illustrates the full reaction cascade together with the probe evolution timeline from FAP-1 to NucRFAP-2.

### 2.2. Hydrazone and Oxime Chemistry: Versatile but pH-Sensitive

Hydrazone-forming probes—where a hydrazine or hydrazide group condenses with an aldehyde to form C=N—are the most widely used class due to well-understood chemistry, broad pH compatibility (pH 5–7), and extensive PET/ICT mechanistic precedent [23,24,31,32,38]. Song and co-workers’ BODIPY-hydrazines [31] and Ding and colleagues’ NIR-emitting hydrazine probe [32] are representative examples demonstrating live-cell applications.

The critical complication is pH-dependence of hydrazone equilibria: both stability constants and formation rates are pH-dependent, meaning probe response shifts substantially between cytoplasm (pH 7.4) and lysosomes (pH 4.5) [23,24]. Selectivity for aldehydes over ketones is also pH-dependent, so selectivity panels conducted at pH 7.4 in PBS may not translate to the actual cellular compartment where the probe accumulates. Reporting probe response versus pH across pH 4.5–8.0 is standard practice that must not be omitted. Hydrazide probes offer improved selectivity at the cost of slower kinetics [34]; oxime-forming probes (hydroxylamine) are the most hydrolytically stable and preferred for analytical or fixed-tissue applications [15].

### 2.3. The BHA Probe: TICT → ICT Mechanism in Detail

**Figure 5:**
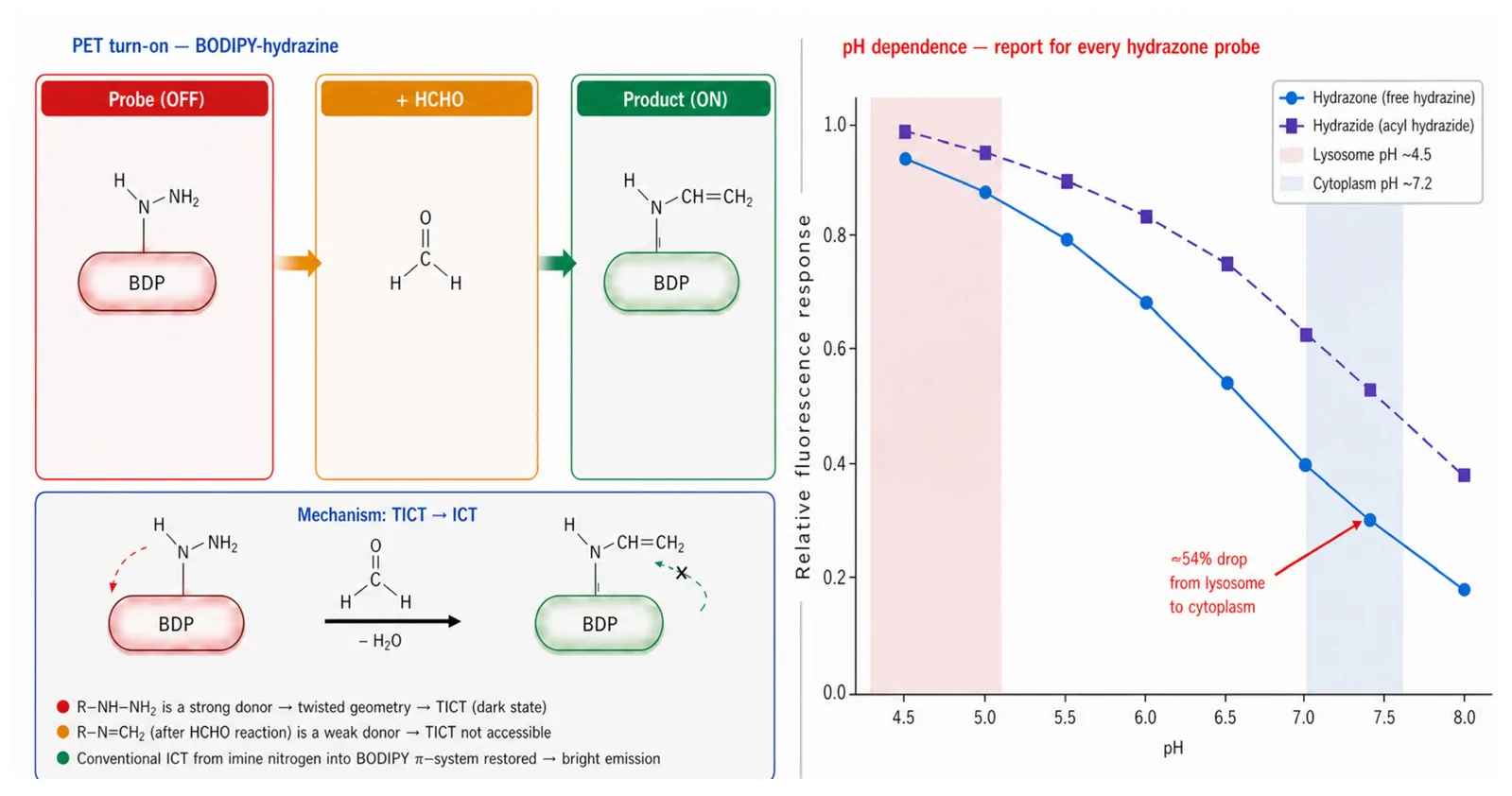
PET mechanism in hydrazine probes and critical pH dependence. (**Left**): PET turn-on via TICT → ICT switching upon hydrazone formation (BODIPY-hydrazine BHA; Song et al. 2018 [31])—free NH-NH_2_ sustains a TICT dark state; condensation with HCHO gives weak-donor N = CH_2_, suppressing TICT and restoring ICT emission. (**Right**): pH-response curves for hydrazone (free hydrazine) and hydrazide (acyl hydrazide) probes across pH 4.5–8.0, showing approximately 54% signal drop between lysosomal (pH 4.5) and cytoplasmic (pH 7.4) conditions—illustrating why pH calibration must be reported for any hydrazone probe intended for compartment-specific imaging. Abbreviations: TICT, twisted intramolecular charge transfer; ICT, intramolecular charge transfer; PET, photoinduced electron transfer. References: [23,24,31,32].

**Figure 6:**
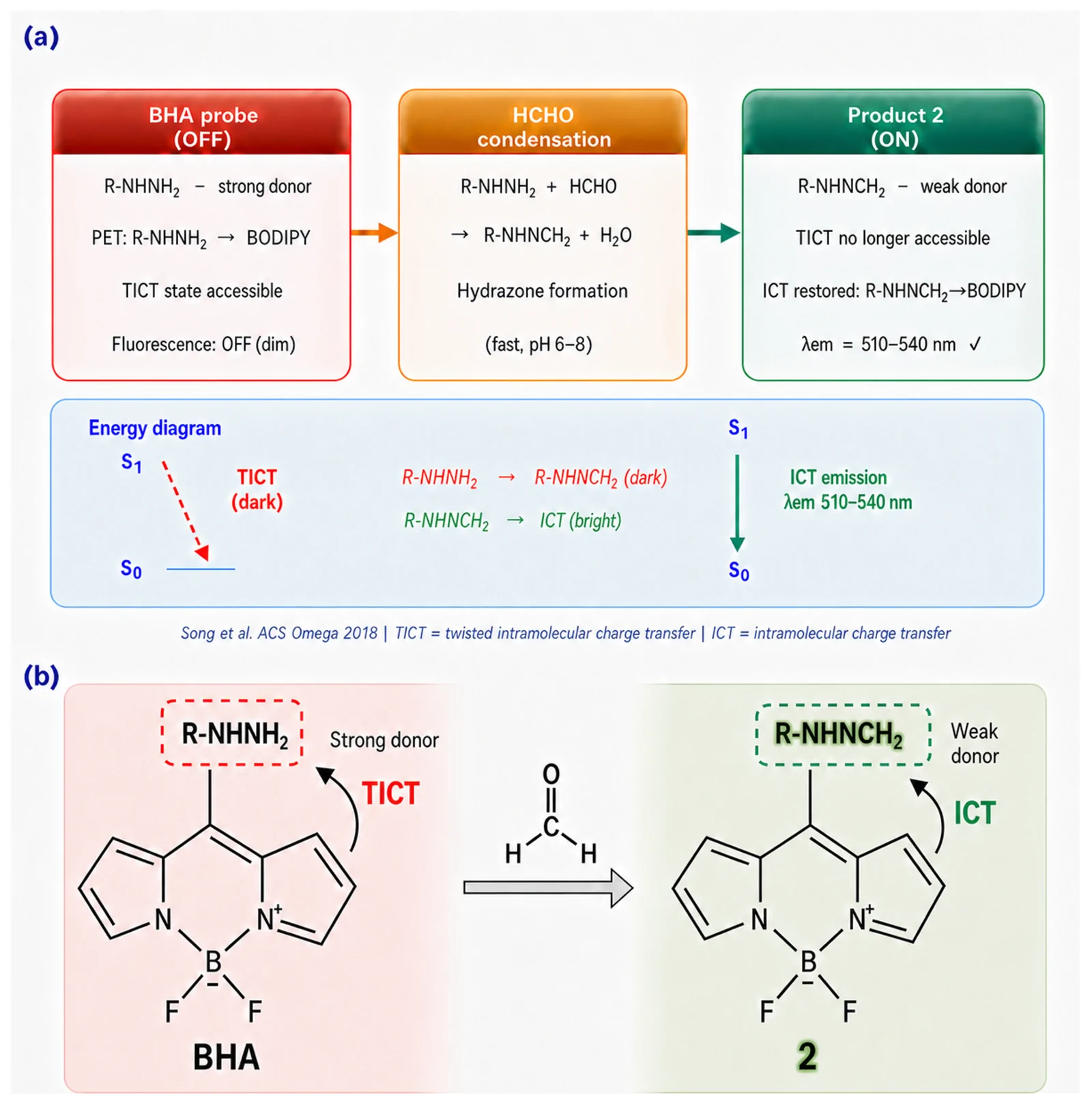
BHA probe TICT → ICT switching for formaldehyde detection (adapted from Song et al. ACS Omega 2018 [31]). (**a**) Mechanism diagram: BHA probe (OFF)—free NH-NH_2_ is a strong electron donor sustaining the TICT dark state, with PET quenching fluorescence; condensation with HCHO converts NH-NH_2_ to weaker-donor N=CH_2_, TICT becomes inaccessible, and conventional ICT emission is restored (λem ≈ 510–540 nm). The energy diagram shows S_1_ deactivation via non-radiative TICT (BHA) versus radiative ICT (product 2). (**b**) Chemical structures of BHA (probe, strong donor NH-NH_2_; TICT active) and product 2 (N=CH_2_ adduct; weak donor; ICT restored; green emission). Dashed boxes highlight the electroactive group in each state. Abbreviation: BHA, BODIPY-hydrazine.

The BODIPY-hydrazine (BHA) probe reported by Song and co-workers is an instructive case study of the TICT → ICT switching mechanism that underlies many hydrazine-type probes [31]. Figure 5 shows the PET turn-on mechanism and the pH-response calibration data that must accompany any hydrazone probe application. Figure 6 shows the BHA mechanism and the original chemical structures from the published work.

### 2.4. Michael Addition: Tailored for Electrophilic Enals

**Figure 7:**
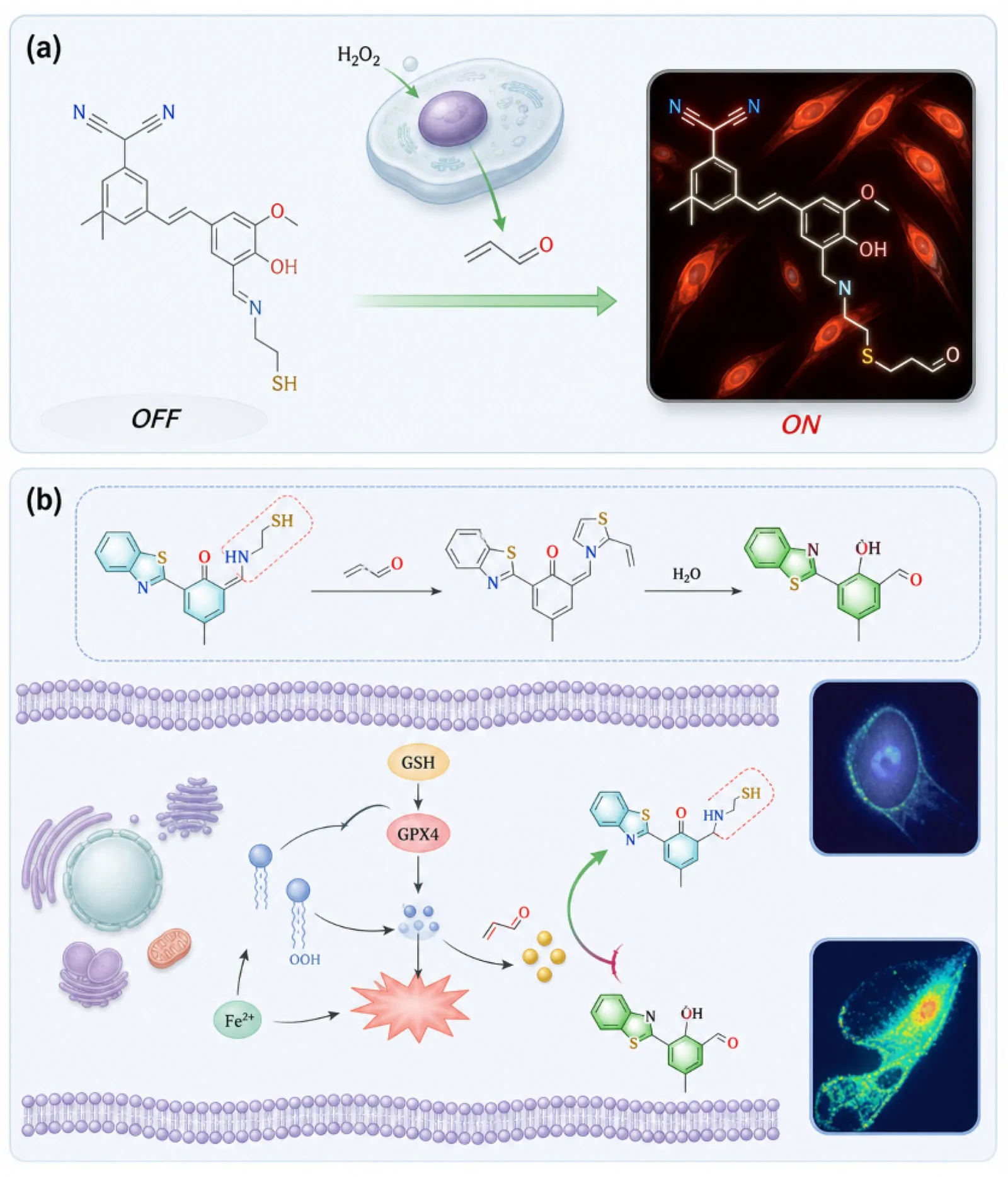
Michael addition probes for α,β-unsaturated aldehydes (enals). (**a**) SWJT-8 probe—H_2_O_2_-induced oxidative stress generates acrolein in cells; the thiol nucleophile undergoes 1,4-addition at the β-carbon to give a red-NIR turn-on product, shown in living cell confocal images [6]. (**b**) HBT-SH ratiometric probe—the first ratiometric acrolein sensor (HBT/ESIPT scaffold); shows the ferroptosis cascade (Fe^2+^-driven lipid peroxidation → GPX4/GSH depletion → acrolein accumulation) and ratiometric cell imaging during ferroptosis (Adapted from Anal. Chem. 2024 [8]). In all enal targets, the conjugated β-carbon (C=C–C=O) is the reactive electrophilic site; saturated aldehydes lack this motif and do not react. Abbreviations: GPX4, glutathione peroxidase 4; GSH, glutathione.

Acrolein, 4-HNE, and related α,β-unsaturated aldehydes are detected by exploiting their conjugated Michael acceptor system: a soft probe nucleophile attacks the β-carbon irreversibly via 1,4-addition, disrupting PET and activating fluorescence [6,7]. Selectivity against saturated aldehydes is intrinsic. They lack the conjugated double bond required for 1,4-addition. Xu and collaborators’ SWJT-8 provides red-NIR turn-on for acrolein [6]; HBT-SH (2024) [8] achieved the first ratiometric acrolein detection and the first direct visualization of acrolein upregulation during ferroptosis, establishing acrolein as a real-time ferroptotic biomarker. Selectivity panels must include 4-HNE, crotonaldehyde, trans-2-hexenal, methylglyoxal, and GSH at 5 mM as a competing nucleophile [6,7]. Figure 7 illustrates the SWJT-8 and HBT-SH probe mechanisms and their cellular validation.

### 2.5. Malondialdehyde Probes

**Figure 8:**
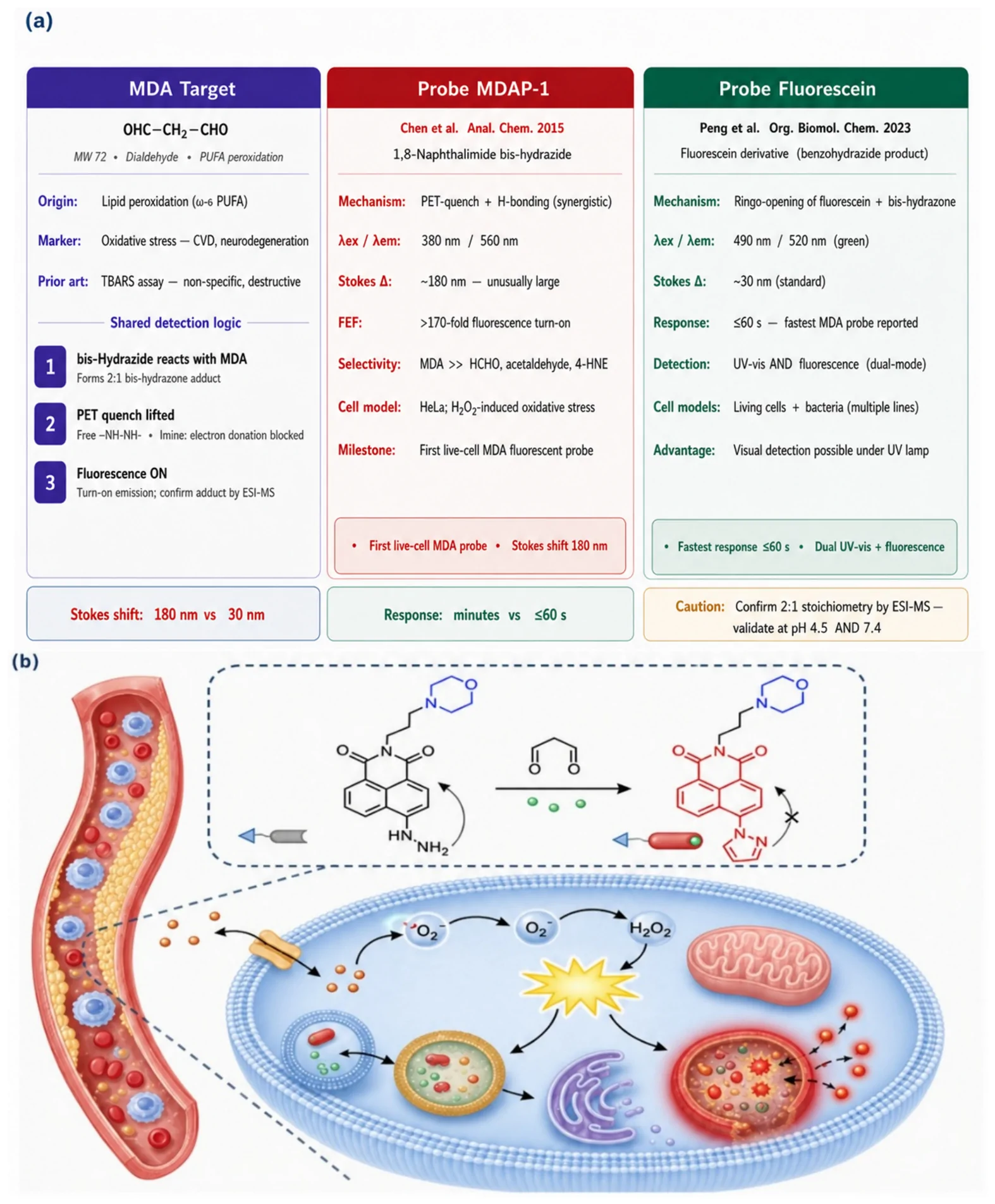
Fluorescent probes for MDA detection. (**a**) MDAP-1 (Chen et al. Anal. Chem. 2015 [9]) vs. fluorescein-based probe (Peng et al. Org. Biomol. Chem. 2023 [10]): side-by-side comparison showing scaffold (1,8-naphthalimide bis-hydrazide vs. fluorescein derivative), mechanism (PET-quench + H-bonding vs. ring-opening + bis-hydrazone), key photophysical parameters (λex/λem 380/560 nm with ~180 nm Stokes shift vs. 490/520 nm), fluorescence enhancement (>170-fold vs. high turn-on), response time (minutes vs. ≤60 s), and cell models. Bottom: comparison of key differentiating parameters and critical caution (confirm 2:1 stoichiometry by ESI-MS; validate probe response at pH 4.5 AND pH 7.4). (**b**) Lysosome-targeted MDA probe for atherosclerosis staging (Adapted from Zhang et al. Chem. Sci. 2025 [11]): naphthalimide scaffold with morpholine lysosome-targeting group reacts with MDA to form a pyrazole adduct blocking PET; macrophage foam cells in ApoE^−/−^ atherosclerotic plaques show stage-dependent lysosomal MDA accumulation correlated with lysosomal dysfunction and the biology invisible to cytoplasmic probes. Abbreviations: FEF, fluorescence enhancement factor; MDA, malondialdehyde; ESI-MS, electrospray ionization mass spectrometry; PET, photoinduced electron transfer; CVD, cardiovascular diseases.

MDA detection evolved from the non-specific TBARS assay to live-cell probes exploiting its unique bifunctionality as a dialdehyde. Chen and co-workers’ MDAP-1 [9] established feasibility through a 2:1 bis-hydrazide adduct mechanism delivering >170-fold fluorescence enhancement and ~180 nm Stokes shift—the first live-cell MDA probe. Peng and colleagues achieved ≤60 s response with a fluorescein scaffold [10] via synergistic ring-opening and bis-hydrazone formation, enabling real-time oxidative burst tracking. Zhang and colleagues’ lysosome-targeted morpholine probe (Chem. Sci. 2025) [11] revealed stage-specific MDA accumulation in the ApoE^−/−^ atherosclerosis model and its biology invisible to cytoplasmic probes. The 2:1 stoichiometry of MDA:probe must be confirmed by ESI-MS before interpreting signal intensity; lysosomal pH (~4.5) shifts hydrazone equilibrium and must be validated separately at pH 4.5 and pH 7.4 [23,24]. Figure 8 compares the MDAP-1 and fluorescein-based probe design alongside the lysosome-targeted probe used in the atherosclerosis model.

### 2.6. Total Aldehyde Sensing via 2-Aminothiophenol Cyclization

**Figure 9:**
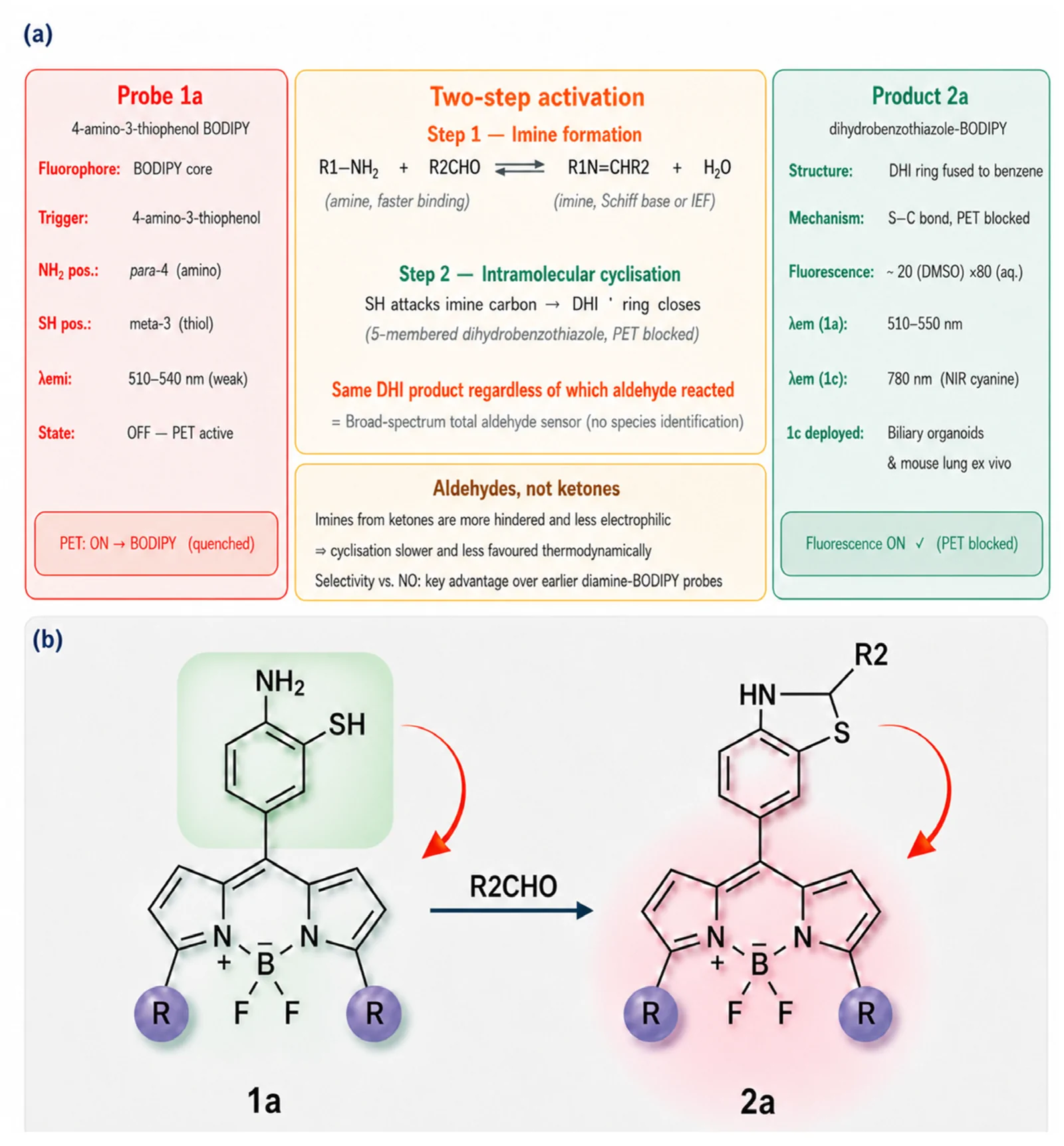
2-Aminothiophenol (2-ATP) platform—total aldehyde sensing. (**a**) Mechanism diagram (Wills, Shirke, Raj et al. Chem. Sci. 2024 [17]): probe 1a (4-amino-3-thiophenol-BODIPY; OFF state, PET active) undergoes two-step activation—Step 1: imine formation (NH_2_ + R-CHO → N=CHR + H_2_O; amine is faster nucleophile than SH at physiological pH); Step 2: intramolecular cyclization (SH attacks imine carbon → 5-membered DHB ring; PET blocked → fluorescence ON, ×28 increase). The same DHB product forms regardless of which aldehyde reacted, making this a total aldehyde sensor with no species discrimination. Product 2a (dihydrobenzothiazole-BODIPY) properties including NIR variant 1c (λem = 780 nm; deployed in biliary organoids and mouse lung ex vivo). (**b**) Chemical structures of probe 1a (2-aminothiophenol trigger highlighted in green) and DHB adduct 2a adapted from Wills et al. [17], showing the S–C bond cyclization that forms the fused dihydrobenzothiazole ring. Abbreviations: 2-ATP, 2-aminothiophenol; DHB, dihydrobenzothiazole; PET, photoinduced electron transfer.

The 2-aminothiophenol (2-ATP) platform (Wills, Shirke, and Raj [17]) provides total carbonyl burden sensing without species identification. The 2-ATP trigger reacts with any aldehyde via imine formation then intramolecular thiol cyclization to give a fluorescent dihydrobenzothiazole (DHB) adduct, with excellent aldehyde/ketone selectivity and no NO cross-reactivity [17]. The modular design accommodates BODIPY, xanthene, or cyanine fluorophores; the NIR cyanine variant (1c, λem = 780 nm) was validated in 3D biliary organoids and ex vivo mouse lungs. Running this platform in parallel with a species-selective probe provides complementary biological context [1,6,17]. Figure 9 illustrates the 2-ATP activation mechanism and the resulting probe and adduct structures.

### 2.7. Amine-Based Probes and Schiff Base Chemistry

Schiff base (imine) formation is the fastest conjugation reaction, but its reversibility is high and hydrolyzes readily at neutral pH, limiting live-cell applicability without NaBH_3_CN reduction [33]. Tan and co-workers showed that NH_2_ position relative to the fluorophore core governs performance: ortho-amine BA-1 (2-(phenylethynyl) aniline) gives LOD of 0.75 µM and <20 min response; meta-BA-2 gives weaker response; and para-BA-3 essentially no response [33]. The geometry–performance relationship is a general design principle: proximal NH_2_ maximizes PET quenching efficiency and hence the fluorescence dynamic range.

The rate column in Table 1 summarizes kinetic data that tracks the intrinsic nucleophilicity of the reactive trigger and the number of bond-forming steps required to reach the fluorescent product, and this hierarchy has direct consequences for which biological processes each chemistry can resolve. Amine/Schiff base condensation is the fastest of all reactions, reflecting the high nucleophilicity of a primary amine attacking the aldehyde carbonyl directly in a single bond-forming step; BA-1 reaches its fluorescence plateau in under 20 min, fast enough to capture acute formaldehyde pulses, but the same kinetic favorability that drives rapid imine formation also favors rapid reverse hydrolysis at neutral pH, so the fast forward rate does not translate into a long-lived signal unless the imine is chemically reduced [23,24,33].

Hydrazine-based hydrazone formation and Michael addition both fall into the “fast” category but for different mechanistic reasons: hydrazine nitrogen is a stronger nucleophile than a simple amine toward carbonyl carbon, giving hydrazone-forming probes (BHA, MDAP-1) response times on the order of seconds to a few minutes [9,23,24,31], while Michael-addition probes (SWJT-8, HBT-SH) achieve comparably fast kinetics because the soft thiol or amine nucleophile attacks an activated β-carbon that is intrinsically more electrophilic than a simple aldehyde carbonyl, with 1,4-addition to acrolein or 4-HNE typically complete within minutes under cellular conditions [6,8]. The 2-aza-Cope/Mannich cascade and the 2-aminothiophenol cyclization are both classified as “moderate” because the rate-limiting step in each case is not the initial nucleophilic attack but a subsequent intramolecular rearrangement or cyclization: the [3,3]-sigmatropic rearrangement in the aza-Cope cascade and the intramolecular thiol attack on the imine carbon in the 2-ATP platform both require a specific molecular geometry to be sampled after the initial, fast condensation step, which adds a kinetic delay of minutes to tens of minutes relative to simple hydrazone or Schiff base formation [1,2,3,4,17].

**Figure 10:**
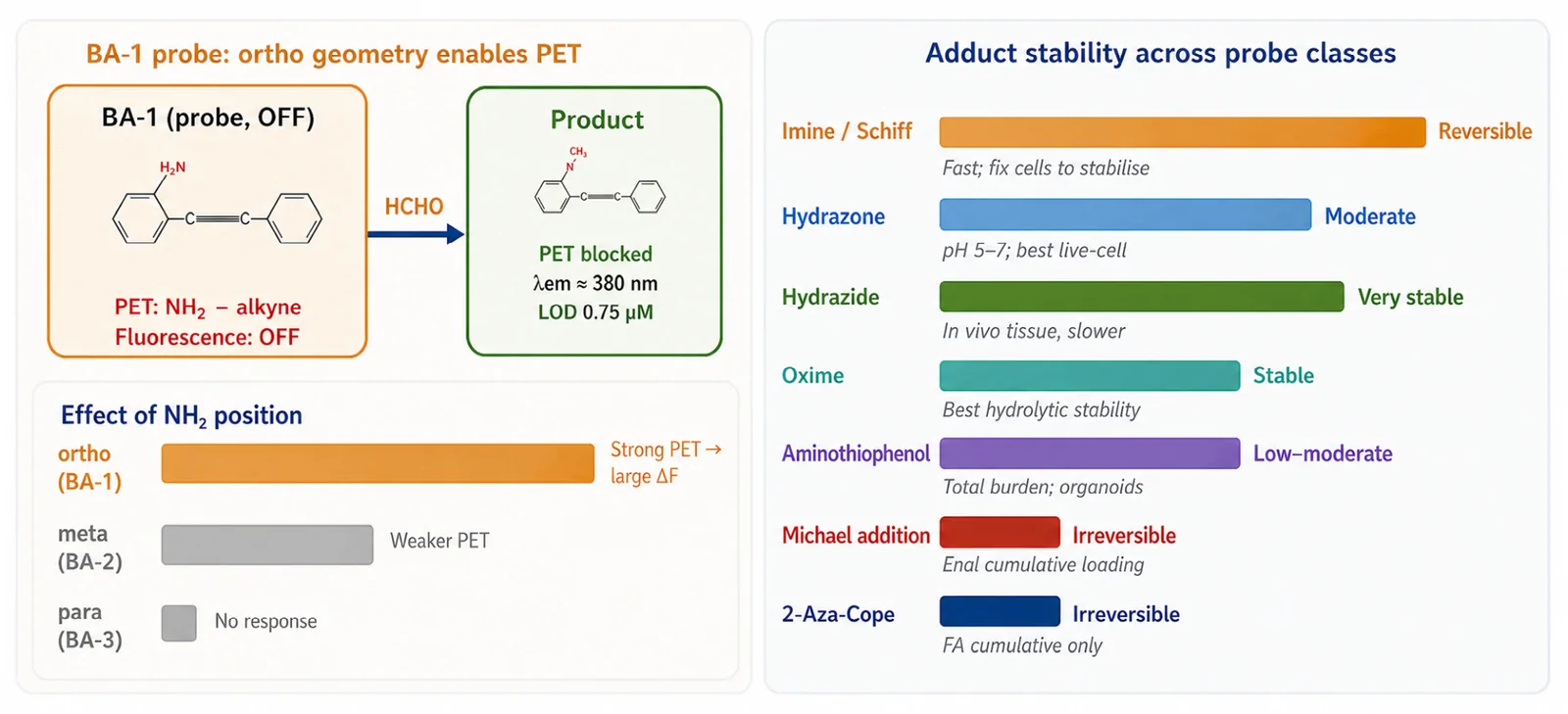
BA-1 Schiff base probe and adduct reversibility comparison across all six probe classes. (**Left**): BA-1 reaction scheme (ortho-NH_2_ + HCHO → N=CH_2_ imine; LOD 0.75 µM) and NH_2_ position effect—ortho (BA-1) >> meta (BA-2) > para (BA-3 ≈ no response), shown as proportional bar chart. (**Right**): ranked horizontal bar chart of adduct stability across all probe classes, from reversible (imine/Schiff: fast but hydrolyzes) through moderate (hydrazone: pH 5–7) to irreversible (2-aza-Cope, Michael addition: cumulative sensing only). Longer bar = more stable adduct = better for cumulative sensing but cannot track real-time aldehyde flux. Abbreviations: LOD, limit of detection; PET, photoinduced electron transfer. References: [23,24,33].

Hydrazide and hydroxylamine (oxime-forming) chemistries are the slowest of the seven, a direct consequence of the reduced nucleophilicity of the acylated nitrogen in hydrazides and the oxygen-centered nucleophile in hydroxylamines relative to a free hydrazine or amine [15,23,24,34]; this same reduced nucleophilicity is what gives these two chemistries their superior selectivity and product stability, illustrating the general rate–selectivity trade-off that runs through the entire table. In practice, this kinetic spread of roughly two orders of magnitude in response time—from sub-minute for Schiff base and Michael addition chemistries to tens of minutes for hydrazide and oxime platforms—means that the choice of conjugation chemistry should be matched not only to the target aldehyde’s identity but to the timescale of the biological process under study: rapid signaling events such as an oxidative burst or an acute demethylation pulse demand the fast end of this range, whereas integrative measurements of cumulative aldehyde exposure in fixed tissue or organoid sections can tolerate, and may benefit from, the slower but more selective bioconjugation chemistries. Figure 10 summarizes the BA-1 probe design and compares adduct reversibility across all conjugation strategies.

### 2.8. Comparative Performance and Probe Selection

Beyond cataloging the available chemistries, it is useful to ask which probe is preferable for a given target and task. For formaldehyde, the 2-aza-Cope probes (FAP-1, FFP706) remain the selectivity benchmark for qualitative live-cell and tissue imaging, whereas the organelle-targeted ratiometric sensors MitoRFAP-2 and NucRFAP-2 are preferred wherever absolute, compartment-resolved quantitation is required; BHA-type BODIPY-hydrazines offer a fast, synthetically simple turn-on option when pH is controlled, and the Schiff-base BA-1 suits rapid transient sensing where in situ reduction is acceptable. For malondialdehyde, the fluorescein probe of Peng et al. is the fastest (≤60 s), MDAP-1 delivers the largest turn-on (>170-fold), and the lysosome-targeted morpholine probe of Zhang et al. is the platform of choice for disease staging. For the α,β-unsaturated aldehydes, the ratiometric HBT-SH is preferable to the turn-on SWJT-8 when quantitation matters, while the 2-aminothiophenol platform is the appropriate choice when total carbonyl burden, rather than species identity, is the question. Table 2 collates the reported photophysical and performance parameters in a common format.

## 3. Photophysical Readout Strategies

Choosing a probe chemistry is the more tractable half of the design problem. The most demanding question and one that receives surprisingly little explicit attention in the primary literature is the final optical signal. An aldehyde has reacted, a fluorophore has switched on, switched off, or shifted in wavelength, and the result is a number on a screen. What that number represents depends entirely on which readout strategy generated it, and this distinction separates a qualitative observation from a measurement that can bear the weight of a quantitative biological claim [30,39,40]. We address the four principal strategies below in order of increasing experimental demand, from the most accessible to the most instrumentally exacting.

### 3.1. Turn-On Intensity Probes

Turn-on probes are the field’s default design: reaction with the target aldehyde relieves a PET or ICT quenching pathway and the fluorophore brightens [31,33]. They are the most synthetically accessible class, which accounts for their predominance. The essential caveat is interpretive. Fluorescence intensity is the product of probe concentration and per-molecule emission efficiency, so any comparison between conditions, such as treated versus untreated or diseased versus healthy, assumes equal probe loading, photobleaching, and optical path length. Those assumptions hold reasonably for a single monolayer imaged under matched conditions, but not in tissue or organoids, where an intensity difference can reflect differential uptake as readily as a difference in aldehyde concentration. Turn-on probes therefore remain the correct first tool for establishing that a probe responds to its target and for semi-quantitative comparison within one controlled session, but an intensity value is not a concentration, which is the most common interpretive overreach in this literature.

### 3.2. Ratiometric Dual-Emission Probes

Ratiometric probes address the loading problem directly by reporting the ratio of two emission intensities, one aldehyde-responsive and one invariant [2,39]. Because both signals arise from the same molecule in the same pixel, factors that affect both channels equally, such as uptake variation, excitation drift, and photobleaching, cancel in the ratio, isolating the aldehyde-dependent chemistry. This converts an experiment that shows that something changed into one that quantifies by how much, making a ratiometric readout close to a requirement whenever conclusions rest on comparing absolute aldehyde levels across cell lines, disease stages, or genotypes. The cost is a second, aldehyde-insensitive channel plus calibration in a matrix approximating the target compartment. MitoRFAP-2 and NucRFAP-2 [3,4] are the current benchmarks, yielding quantitative, subcellular-resolution formaldehyde maps that intensity-only probes cannot provide.

### 3.3. Fluorescence Lifetime Imaging Microscopy (FLIM)

Where ratiometric design solves the loading problem by introducing a reference channel, fluorescence lifetime imaging solves it by changing the underlying observable entirely. The fluorescence lifetime, τ, is the average duration a fluorophore remains in its excited state before returning to the ground state and this is an intrinsic molecular property, independent of fluorophore concentration, excitation intensity, or the extent of photobleaching [39]. Two pixels of markedly different intensity can share an identical lifetime if their fluorophore environment is equivalent; conversely, two pixels of equal intensity can exhibit markedly different lifetimes if the underlying chemistry differs. For aldehyde probes whose reaction with the target analyte alters the excited-state deactivation pathway—the TICT-to-ICT transition accompanying hydrazone formation, discussed throughout this review, is the representative case—the resulting lifetime shift provides a concentration-independent measure of reaction progress at each pixel, regardless of local probe abundance.

This property is particularly valuable in thick tissue, where probe distribution is rarely uniform and where intensity imaging alone cannot distinguish “more probe here” from “more reaction here.” The principal limitation is instrumental: FLIM requires time-correlated single-photon counting or frequency-domain detection hardware beyond a standard confocal configuration, with correspondingly longer acquisition times. For investigations in which the biological question demands absolute quantification rather than relative trends, this investment is justified, and we would extend the point further—FLIM and ratiometric design are not competing approaches but complementary ones. A probe offering both readouts simultaneously provides two independent, internally consistent measurements capable of validating one another.

### 3.4. Two-Photon Excitation

The preceding strategies concern how a collected signal should be interpreted. Two-photon excitation addresses an earlier and distinct problem: how deep into a sample a usable signal can be generated at all. Conventional single-photon excitation relies on visible-wavelength light, which scatters extensively in tissue and is absorbed by hemoglobin, melanin, and other endogenous chromophores within the first several tens of microns. Two-photon excitation circumvents this limitation by employing near-infrared light (700–1000 nm, within the range relevant to this review) at sufficient intensity that a fluorophore is excited through the near-simultaneous absorption of two lower-energy photons rather than a single higher-energy photon [35,36]. Because this process requires a high local photon density, it occurs efficiently only at the laser’s tight focal point, which confines both excitation and the associated photodamage to a small focal volume rather than the full illumination cone. The practical benefit is substantial: submicron resolution at depths of hundreds of microns, in tissue that would otherwise scatter visible light into uselessness within the first cell layer.

Not every fluorophore scaffold is well suited to two-photon excitation, and this is where probe chemistry and photophysics must be considered jointly rather than in isolation. The governing parameter is the two-photon absorption cross-section, σ_2_, expressed in Goeppert–Mayer units (GM); below approximately 100 GM, the resulting signal is too weak for practical imaging even with high laser power, requiring scaffolds to be screened or purpose-engineered with this constraint in mind. The ER-targeted two-photon formaldehyde probe discussed elsewhere in this review [37] illustrates the value of this approach with particular clarity: by combining two-photon excitation with subcellular targeting, it achieved direct imaging of formaldehyde-induced endoplasmic reticulum stress in intact neural tissue—a result that, to our knowledge, no single-photon probe has reproduced. This outcome was only possible once the depth-penetration and subcellular-resolution problems were solved jointly, and it serves as a useful reminder that the four readout strategies discussed in this section are not mutually exclusive alternatives but components of a single toolkit, to be combined according to the demands of the biological question under investigation.

## 4. Applications Across Biological Scales

**Figure 11:**
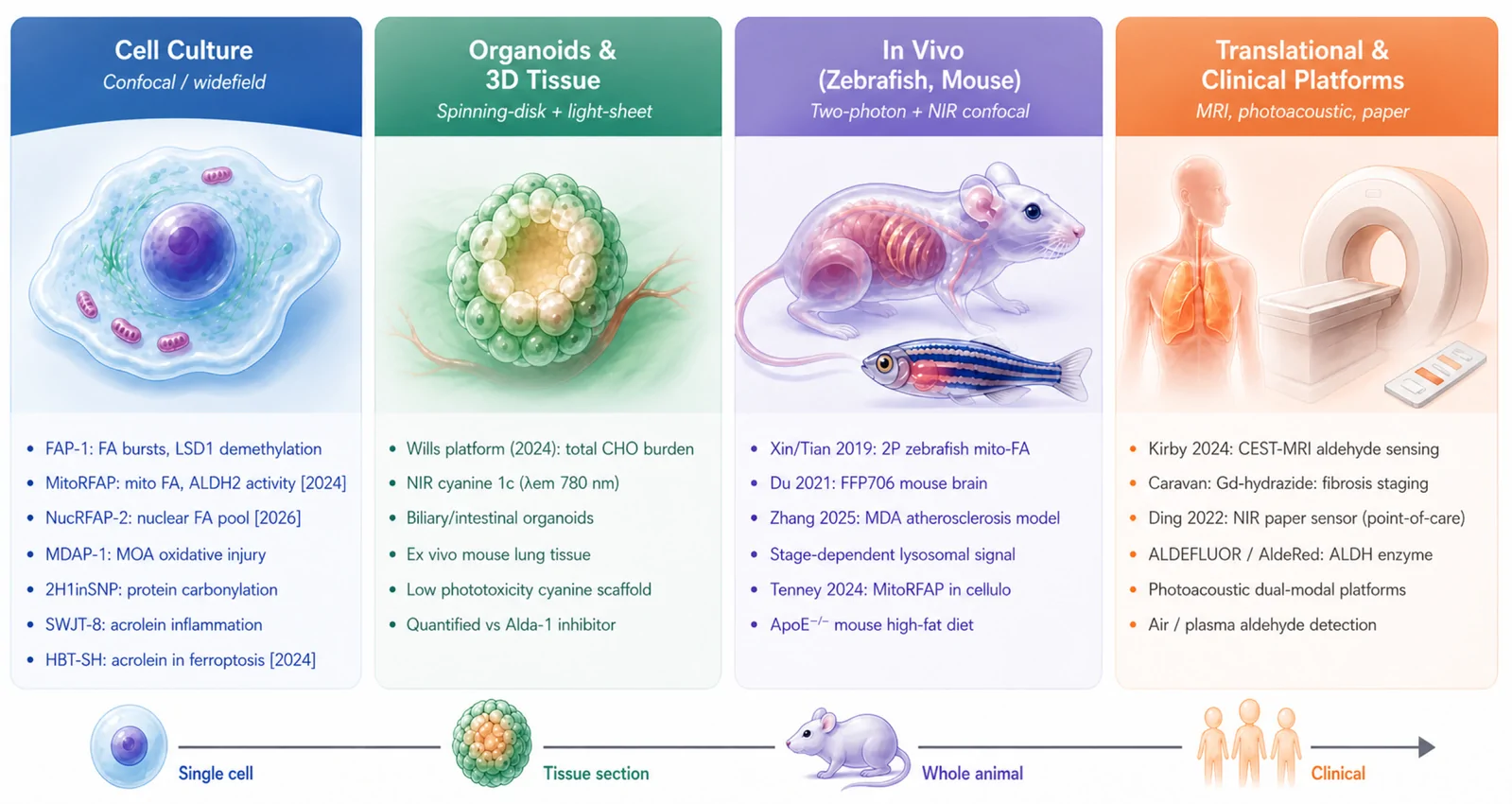
Aldehyde probe applications organized by biological scale. (**Left**): Cell culture (confocal/widefield), key probes at single-cell resolution including FAP-1 (FA/LSD1), MitoRFAP (mito FA), MDAp-1 (MDA), SWJT-8 and HBT-SH (acrolein/ferroptosis), and 2Hzin5NP (protein carbonyls). (**Center-left**): Organoids and 3D tissue (spinning-disk + light-sheet). (**Center-right**): In vivo models in zebrafish and mouse (two-photon and NIR confocal). (**Right**): Translational and clinical platforms (MRI, photoacoustic, point-of-care). Bottom axis: increasing biological complexity and imaging depth from single cell to clinical. References: [1,2,3,4,6,8,9,10,11,16,17,20,32,34,36].

The translation of probe chemistries and photophysical strategies into genuine biological insight has progressed systematically from single-cell proof-of-concept experiments to quantitative in vivo disease staging. Figure 11 provides a scale-organized overview of the major results [1,2,3,4,6,8,9,10,11,16,17,25,34,35,36,38].

### 4.1. Single Cells

**Figure 12:**
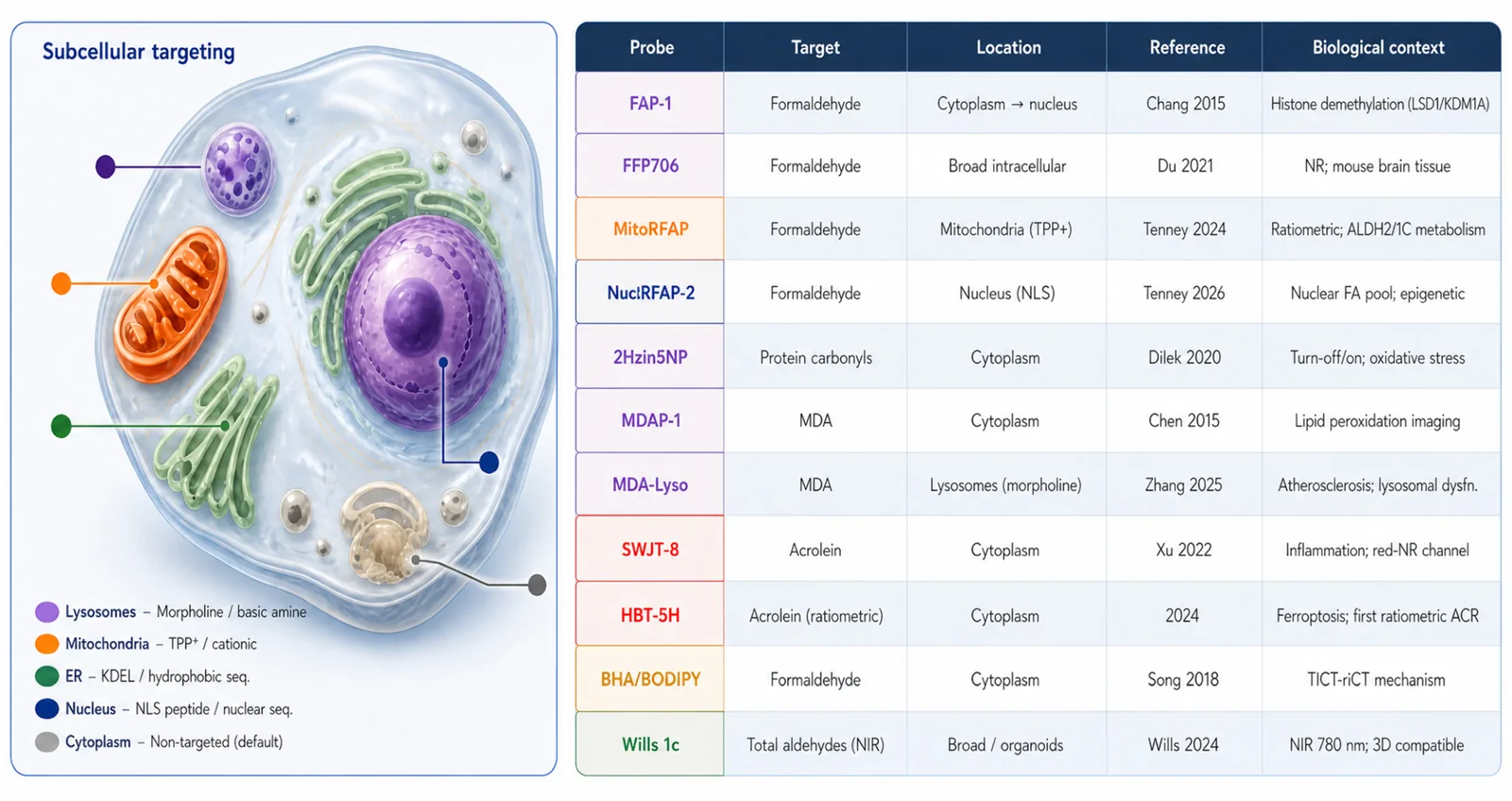
Cellular aldehyde imaging: subcellular targeting strategies and probe reference catalog. (**Left**): Cell schematic showing established organelle-targeting motifs—lysosomes (morpholine/basic amine), mitochondria (TPP^+^/cationic), endoplasmic reticulum (KDEL/hydrophobic sequence), nucleus (NLS peptide), cytoplasm (non-targeted default). (**Right**): Probe catalog table listing probe name, target species, subcellular location, key reference, and biological context. References: [1,2,3,4,6,8,9,10,11,17,20,31,37].

FAP-1’s demonstration that LSD1/KDM1A demethylase activity produces detectable FA transients in living breast cancer cells [1] reframed formaldehyde as a process-coupled metabolite reporting on chromatin remodeling in real time. Subsequent work established FA as a 1C signaling molecule connecting LSD1-dependent histone demethylation to nucleotide biosynthesis [1,2,3,4,5]. Chen’s MDAP-1 [9] and Peng’s fluorescein-MDA probe [10] established live-cell MDA tracking at oxidative-burst time resolution; SWJT-8 [6] and HBT-SH [8] provided acrolein coverage for inflammatory and ferroptotic models; our 2Hzin5NP probe added total protein carbonylation imaging [20]. Figure 12 summarizes the probe catalog with subcellular targeting assignments.

### 4.2. Organoids and Three-Dimensional Tissue Models

Moving to 3D models introduces imaging challenges that cannot be treated as scaled-up 2D protocols: light scattering increases steeply with depth, probe loading becomes spatially heterogeneous, and photodamage risks increase in metabolically active 3D structures. Wills and colleagues engineered NIR probes specifically for 3D compatibility and validated them in biliary organoids and ex vivo mouse lung with pharmacological controls (Alda-1 inhibition, ethanol treatment) confirming biological specificity [17,41]. Spinning-disk confocal and light-sheet microscopy are preferred over point-scanning CLSM for reduced phototoxicity in 3D cultures [17,30,42].

### 4.3. In Vivo and Ex Vivo Imaging

**Figure 13:**
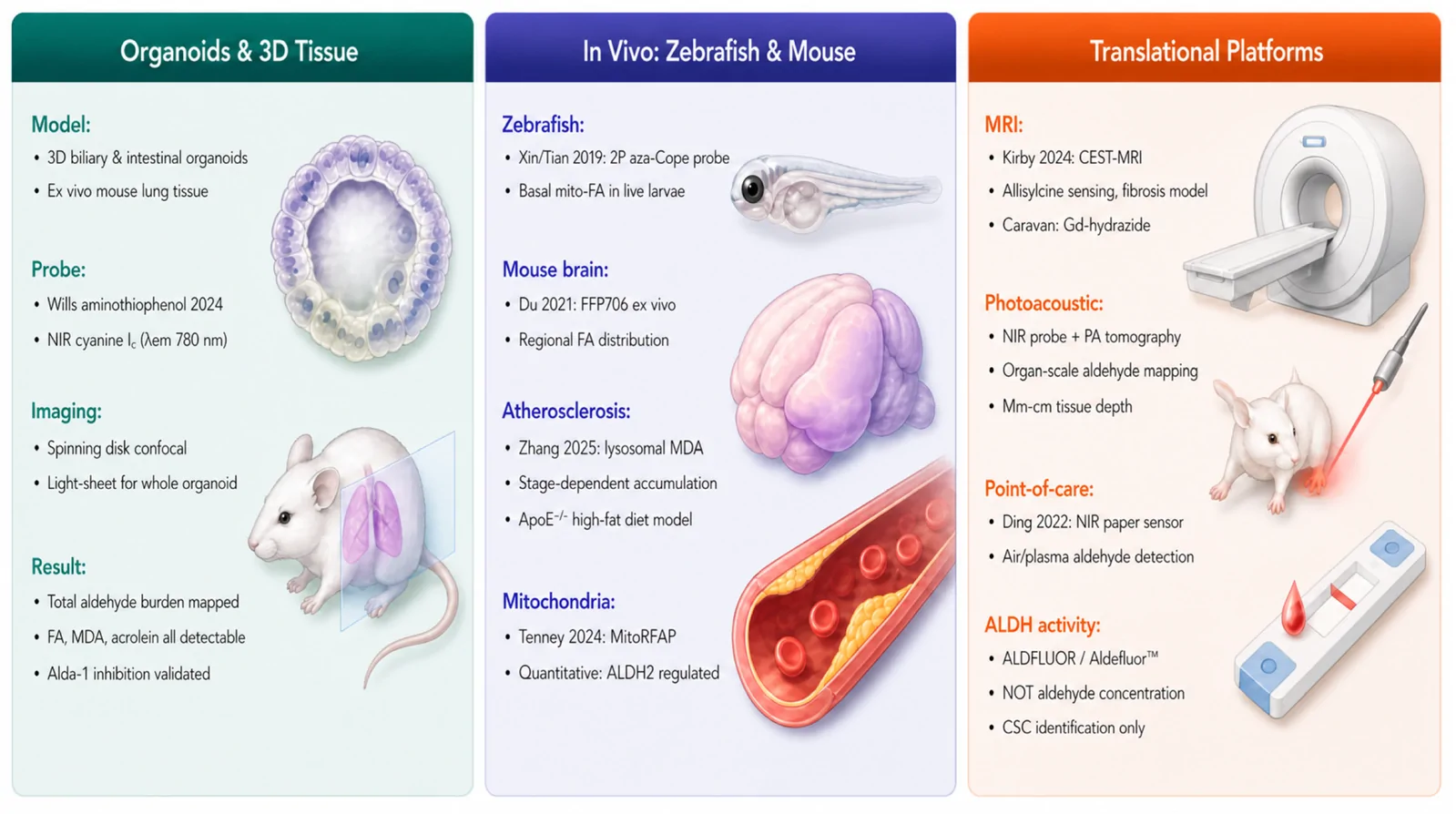
Aldehyde probe deployment beyond cell culture: organoids, in vivo models, and translational platforms. (**Left**): Organoids and 3D tissue—Wills et al. 2024 aminothiophenol platform [17] in biliary/intestinal organoids and ex vivo mouse lung; NIR cyanine 1c (λem = 780 nm); spinning-disk confocal and light-sheet imaging modalities. (**Center**): In vivo zebrafish and mouse models—zebrafish (Xin/Tian 2019 [36], 2P basal mito-FA); mouse brain (Du 2021 [2], FFP706 regional FA); atherosclerosis (Zhang 2025 [11], lysosomal MDA, ApoE^−/−^ model); mitochondria (Tenney 2024 [3], MitoRFAP quantitative ratiometric). (**Right**): Translational platforms—MRI (Kirby 2024 [25] CEST-MRI; Caravan [34] Gd-hydrazide fibrosis); photoacoustic (NIR + PA tomography; organ-scale depth); point-of-care (Ding 2022 [32] NIR paper sensor); ALDH activity (ALDEFLUOR/AldeRed™ [13,16]—reports enzyme activity, NOT free aldehyde concentration). References: [2,3,8,11,13,16,17,25,32,34,36].

Xin and Tian’s two-photon visualization of basal mitochondrial FA in live zebrafish larvae [36] provided direct in vivo evidence for constitutive 1C metabolic activity. Du’s FFP706 revealed regional FA distribution in ex vivo mouse brain [2]. MitoRFAP-2 provided quantitative ratiometric data showing ALDH2 activity as the dominant determinant of steady-state mitochondrial FA levels [3]. Zhang and colleagues’ lysosome-targeted MDA probe in ApoE^−/−^ mice revealed stage-specific lysosomal MDA accumulation correlated with plaque histology—the most compelling disease-staging result in this literature [11]. Figure 13 summarizes these organoid, in vivo, and translational deployments.

### 4.4. ALDH Activity Probes Versus Aldehyde Concentration Sensors

ALDH activity probes (ALDEFLUOR, AldeRed™ 588-A [13,16]) deliver a fluorescent aldehyde substrate that ALDH enzymes oxidize to a retained fluorescent acid product. The signal reports enzyme activity—not free aldehyde concentration. A cell with high ALDH1A1 expression may show a strong ALDEFLUOR signal while showing minimal signal from an aldehyde-sensing probe, because the high enzyme activity efficiently clears free aldehyde. Both readings are simultaneously correct. ALDH activity probes are appropriate for identifying ALDH-high subpopulations (cancer stem cell biology) or quantifying enzyme activity differences. They must never be described as measurements of free aldehyde concentration [13,16]. Running both classes in parallel—aldehyde-sensing and ALDH activity—gives a mechanistically complete picture that neither class can provide alone.

## 5. Critical Challenges

The following challenges constrain progress across the field and remain incompletely solved. Table 3 provides a structured overview; the three most important are expanded below.

### 5.1. Selectivity Is More Difficult than Most Selectivity Panels Acknowledge

Most published selectivity panels are conducted under conditions that do not reflect the intracellular environment and therefore do not adequately validate the biological claims they support [1,7,24]. Demonstrating selectivity at pH 7.4 in PBS does not establish selectivity in a cytoplasm containing mM GSH, 200 µM cysteine, trace methylglyoxal, and structurally similar competing aldehydes. Minimum standards: all structurally related aldehydes at physiological concentrations; relevant ketones; GSH at 1–5 mM; H_2_O_2_ at 100 µM; measurements at pH 7.4 and pH 5.0–5.5 for lysosomal-application probes; quantitative cell viability assay at the imaging concentration. The 2025 Semwal/Das RSC Advances review [18] and Jana 2022 Chemistry—Asian Journal review [43] provides useful frameworks for selectivity evaluation.

### 5.2. Quantitative Imaging in Tissue Remains Unsolved

Probe loading varies with depth and tissue architecture, scattering modifies excitation and emission path lengths, and photobleaching rates differ across the imaging volume [39]. Most tissue imaging papers present spatial distribution maps but cannot report absolute aldehyde concentrations since it is very challenging. Ratiometric probes and FLIM address this in principle; for most aldehyde classes other than FA, ratiometric probes with adequate photophysical characterization for quantitative tissue imaging do not yet exist [2,3,39]. This gap is the principal factor limiting probe translation to disease-staging tools.

### 5.3. Real-Time Aldehyde Flux Measurement Remains Inaccessible

Every reaction chemistry in Section 2 produces an irreversible or near-irreversible adduct, meaning all existing probes integrate aldehyde exposure over time rather than reporting instantaneous concentration [23,24]. The oxime/hydroxylamine equilibrium is the most promising starting point for reversible sensing: oximes are genuinely more reversible than hydrazones at physiological pH and their equilibrium position can be tuned by systematic probe structure modification [15,23,24]. This is identified as the highest-priority open problem in the field.

### 5.4. Current Approaches to Reversible Sensing and Their Limitations

Because every chemistry in Section 2 forms an effectively irreversible adduct, genuinely reversible detection requires a recognition reaction that behaves as a true equilibrium on the imaging timescale. Three chemical strategies are currently credible, each with a characteristic limitation. (i) Oxime formation from a hydroxylamine trigger is the most reversible of the condensation chemistries at physiological pH, and its equilibrium position can be tuned through the electronics and sterics of the hydroxylamine partner [15,23,24]; the limitation is that the reduced nucleophilicity permitting reverse hydrolysis also slows the forward reaction, so response time lengthens and signal amplitude falls as reversibility is increased. (ii) Nucleophilic catalysis of hydrazone/oxime exchange, for example by aniline or substituted anilines acting as transimination catalysts, accelerates equilibration and is well established for making hydrazone/oxime linkages dynamic in bioconjugation [23,24]; the limitation for live-cell use is that the catalyst must reach an effective intracellular concentration without perturbing the system, which has not been demonstrated for an aldehyde-imaging probe. (iii) Ortho-Boronic-acid-assisted iminium/oxime formation, in which a neighboring boronic acid stabilizes the reversible C=N adduct, both speeds exchange and lowers the effective equilibrium concentration [44,45,46,47]; the limitation is competing boronate reactivity toward diols and H_2_O_2_ that can erode selectivity in a cellular matrix [48].

The central design tension is therefore intrinsic rather than incidental: the features that make an adduct reversible, namely a weaker nucleophile, catalyzed exchange, or a stabilized but labile C=N bond, are the same features that erode the signal-to-noise advantage of an accumulating, irreversible probe. To our knowledge, no reversible, aldehyde-selective fluorescent probe has yet been validated in living cells and identifying an oxime- or boronic-acid-assisted equilibrium that sits usefully in the nanomolar-to-micromolar range while retaining selectivity against glutathione and competing carbonyls remains the highest-priority open problem in the field. It is also a well-defined target for the computational triage described in Section 6.4, since a substantial published record of oxime and hydrazone equilibrium constants already exists on which a predictive model could be trained.

## 6. Directions for Future Development

Every field eventually reaches a point where the foundational experiments have been done and what remains are the harder problems that were visible all along. Aldehyde imaging has reached that point. The four directions outlined below share three properties that, in our view, justify sustained investment: each has a clear and testable definition of success, each build on chemistry or instrumentation already validated in adjacent fields, and each would open biological questions that are presently inaccessible by any existing method.

### 6.1. NIR-II Fluorophores for Deep-Tissue Imaging

The case for this direction stays on a single physical relationship: tissue scattering decreases approximately with the inverse fourth power of wavelength. This relationship has driven the field’s steady progression toward red and near-infrared fluorophores over the past two decades, and it points clearly to where the next gain lies. The NIR-II window—approximately 1000 to 1700 nm—offers a substantial further reduction in both scattering and endogenous autofluorescence relative to the NIR-I region occupied by most current probes, and whole-organ imaging at millimeter depth in live rodents is now practically achievable with InGaAs detectors that have moved from specialized equipment to standard laboratory instrumentation [26,27]. No aldehyde-responsive probe has yet been demonstrated in this window, and the reason is a genuine synthetic obstacle rather than a lack of motivation: extending emission past 1000 nm in an organic small molecule typically requires extending π-conjugation, which tends to bring with it poor aqueous solubility, aggregation, and large planar scaffolds that resist functionalization with an aldehyde-reactive trigger without disturbing the electronic structure responsible for the red-shifted emission in the first place.

We see two scaffold families positioned to resolve this tension. Aggregation-induced emission (AIE) systems convert what is normally a liability—aggregation—into the source of the fluorescence signal itself, sidestepping the solubility problem rather than fighting it. Heptamethine cyanines, already dominant in NIR-I clinical and preclinical imaging, have recently been shown to tolerate substitution at the central methine position in ways that tune photostability and solubility without collapsing the chromophore [49]—precisely the kind of synthetic handle needed to install a 2-ATP or hydrazide trigger. A heptamethine scaffold carrying one of these established, modular trigger chemistries represents, in our assessment, a near-term and achievable target for a synthetic group with cyanine experience, not a multi-year basic research program.

### 6.2. Reversible Probes for Real-Time Flux Measurement

Every probe chemistry discussed in Section 2 forms an adduct that, once formed, does not dissociate on any biologically relevant timescale. This irreversibility is, in one sense, a design strength: it is precisely what gives these probes their favorable signal-to-noise characteristics, since the product accumulates rather than re-equilibrating away. It is also a fundamental constraint on what these probes can measure. An irreversible probe can answer the question of how much aldehyde a cell has accumulated since the probe was introduced; it cannot answer the question of what the aldehyde concentration is at this specific moment. For a substantial fraction of the most biologically interesting questions in this space, the second question is the one that matters. A formaldehyde transient accompanying a single histone demethylation event, an acrolein spike at the leading edge of an inflammatory response, the real-time escalation of lipid peroxidation as a cell commits to ferroptosis—none of these dynamic processes can be resolved by a probe that only accumulates signal [23,24].

What the field needs is a sensor that behaves the way a calcium indicator behaves: a signal that rises and falls with the underlying analyte concentration because the reaction generating that signal is a true equilibrium rather than a one-way trap. Among the chemistries already available, oxime formation between hydroxylamine and aldehyde stands out as the most chemically credible starting point, since oximes are genuinely more reversible than hydrazones under physiological conditions, and the position of that equilibrium can in principle be tuned through the electronic and steric properties of the hydroxylamine partner [15,23,24]. This will not be a small undertaking—identifying an equilibrium that sits usefully within the nanomolar-to-micromolar range relevant to endogenous aldehyde signaling, while retaining adequate selectivity, is a genuine problem in physical organic chemistry rather than a routine engineering task. It is, however, a problem well suited to computational triage: a substantial published record of oxime and hydrazone formation kinetics and equilibrium constants across diverse aldehyde structures already exists, and a model trained on this record could meaningfully narrow the structural search space before any compound is synthesized.

### 6.3. Integration with Photoacoustic and MRI Platforms

Fluorescence imaging, regardless of how far its wavelength or excitation strategy is pushed, carries an inherent depth ceiling. Even with NIR-II emission and two-photon excitation, fluorescence microscopy remains, at best, a technique for the outer few millimeters of tissue, and for most probes discussed in this review the practical working depth is measured in hundreds of microns. This is entirely adequate for single-cell and organoid biology. It is not adequate for whole-organ aldehyde mapping in a living animal or for any realistic path toward clinical application, and at that point the relevant question shifts from how to improve fluorescence probes to what other detection physics the same recognition chemistry can be coupled to.

Photoacoustic imaging is the most direct answer available to this field, since the underlying physics requires minimal modification of existing probe designs: a chromophore absorbs pulsed near-infrared light, the resulting localized heating generates an ultrasonic pressure wave, and that wave is detected at centimeter depth with sub-millimeter resolution. Any NIR-absorbing aldehyde probe is, in principle, already a candidate photoacoustic contrast agent, since the same absorption event that would normally be followed by emission can instead, or in addition, be read out acoustically, with no change required to the aldehyde-recognition chemistry itself. MRI provides an even more striking demonstration of how transferable this molecular recognition chemistry can be. The Caravan group’s gadolinium-hydrazide probe, built on the same hydrazide chemistry discussed throughout Section 2 and designed to react with allysine residues in fibrotic collagen, was converted into an MRI contrast agent capable of producing quantitative, organ-scale maps of fibrotic burden that correlate with histology in living animals [34], a principle reinforced across a broader set of examples in Kirby and colleagues’ review of MRI-active aldehyde sensors [25]. The clear next objective is a single-probe architecture that does not require a choice between scales—one molecule carrying both a fluorescent reporter for cellular-resolution imaging and a photoacoustic or paramagnetic handle for organ-scale mapping, allowing the same underlying chemistry to identify which cells are active and, within the same animal, where in the body that activity is concentrated.

### 6.4. Computational Tools and Machine Learning in Probe Design

Computational methods have already begun to reshape fluorophore design broadly, predicting absorption and emission wavelengths from structure, screening virtual libraries against multiple design criteria simultaneously, and narrowing the field of synthetic candidates before a single reaction is run [40]. That this progress has not yet extended meaningfully to aldehyde-reactive probes is a consequence of training data, not of method: the large fluorophore property datasets underlying these models were not constructed with aldehyde-responsive trigger chemistry in mind, so a model asked to evaluate a novel trigger–fluorophore combination is extrapolating well beyond its training distribution—precisely the regime in which such predictions are least reliable.

This is a solvable problem, but solving it requires something the field has not yet organized around: a shared data culture. Every laboratory developing a new aldehyde probe generates information of value to the entire community—selectivity panels evaluated under physiologically relevant conditions, response kinetics, photophysical characterization before and after reaction, and, where available, in-cell or in vivo performance data. At present, this information is dispersed in inconsistent formats in the scientific literature, which is not easily accessible to anyone attempting to build a predictive model from the literature. The infrastructure required to address this already exists; platforms such as ChEMBL and established open standards for fluorescence data are mature and actively maintained, so the limiting factor is not technological capability but practice. What is needed is a norm, ideally supported by journals and funding agencies, of depositing probe characterization data in structured, machine-readable form as standard practice.

## 7. Conclusions

A decade ago, the question driving this field was simple: could we even see these small molecules in a living cell? Today that question is settled, and the conversation has moved onto precision, to depth, to whether we can trust what we are measuring. That shift reflects real progress. Probes built on aza-Cope chemistry now report formaldehyde with a selectivity that comes from the reaction mechanism itself, not from simple tuning. Broad-spectrum 2-ATP platforms map total carbonyl burden through entire 3D organoids.

Lysosome-targeted MDA probes have revealed disease-stage-specific biology that no bulk assay could have shown. Organelle-targeted ratiometric sensors give us quantitative formaldehyde maps at subcellular resolution, the first ratiometric acrolein probe has opened a window onto ferroptosis as it happens, and two-photon probes targeted to the ER can monitor aldehyde-driven stress unfold in real tissue. None of this was possible ten years ago. Quantitative imaging deep in tissue, at the resolution disease staging demands, remains out of reach. Selectivity claims in the literature are too often tested under conditions that do not resemble a living cell. And the distinction between an aldehyde-concentration probe and an ALDH activity probe—two fundamentally different measurements—still are not easily interpreted.

These are the critical challenges with visible shapes, and that is itself the measure of how far this field has come. What changes the timeline for closing them is computation. Probe development has, until now, largely proceeded by intuition and iteration—synthesize, test, adjust, repeat. Machine learning models trained on the kinetic and photophysical data already scattered across this literature could turn that loop around: screening candidate trigger–fluorophore combinations for selectivity and reactivity before a single compound is made and narrowing years of synthetic exploration into a shortlist worth pursuing.

## Figures and Tables

**Table 2:** Comparative performance benchmarks for representative aldehyde probes discussed in this review. λex/λem, excitation/emission maxima; SiR, silicon rhodamine; ESIPT, excited-state intramolecular proton transfer; NR, not reported. Limits of detection are omitted because the primary sources determine them under heterogeneous buffer, pH, and instrument conditions and the values are therefore not directly comparable across probes; individual LOD values are quoted in the text where the original report provides them. References: [1,2,3,4,6,8,9,10,11,17,31,33].

Target	Representative Probe (Refs)	λex/λem (nm)	Response Time	Selectivity/Best Use
FA	FAP-1 [1]	–/≈720 (SiR)	moderate	FA-selective (aza-Cope); live-cell/LSD1 imaging
	FFP706 [2]	≈650/720	moderate	SiR dye; validated ex vivo in mouse brain
	MitoRFAP-2/NucRFAP-2 [3,4]	ratiometric (dual-emission)	moderate	Organelle-targeted, compartment-resolved FA
	BHA (BODIPY-hydrazine) [31]	≈510–540 em	fast (s to min)	Broad carbonyl turn-on; pH-sensitive
	BA-1 (Schiff base) [33]	≈380 em	<20 min	Fast, reversible; needs NaBH_3_CN fixation
MDA	MDAP-1 [9]	380/560 (~180 nm Stokes)	minutes	>170-fold turn-on; 2:1 stoichiometry
	Fluorescein-MDA [10]	490/520	≤60 s	Fastest MDA probe; dual UV-vis/fluorescence
	Lyso-morpholine probe [11]	NR	NR	Lysosome-targeted; ApoE^−/−^ atherosclerosis
Acrolein/enals	SWJT-8 [6]	red–NIR	minutes (1,4-addition)	Enal-selective; H_2_O_2_ oxidative-stress model
	HBT-SH [8]	ratiometric (ESIPT)	minutes	First ratiometric acrolein probe; ferroptosis
Total aldehyde	2-ATP 1a/1c [17]	510–550/780 (1c)	moderate	Pan-aldehyde; ×28 turn-on; organoids/ex vivo lung

**Table 3:** Main challenges in aldehyde probe development and current mitigation strategies. FA = formaldehyde; GSH = glutathione; AIE = aggregation-induced emission; ESIPT = Excited-State Intramolecular Proton Transfer; FLIM = Fluorescence-lifetime imaging microscopy. References: [1,6,7,23,24,27,30,31,33,39].

Challenge	Primary Issue	Underlying Cause	Imaging Impact	Refs	Best Current Mitigation
Selectivity	Aldehyde cross-reactivity	Similar carbonyl electrophilicity	Off-target labeling	[1,7,24]	2-Aza-Cope for FA; ESIPT for acrolein
pH sensitivity	Variable response	Hydrazone equilibrium. pH-dep.	Non-reproducible compartment data	[23,24]	Calibrate across pH 4.5–8.0 as standard
Competing nucleophiles	GSH quenching	mM cytoplasmic thiols	Underestimate of enal burden	[6,7]	Test all probes at 5 mM GSH
Quantitation in 3D	Intensity vs. depth	Scattering; heterogeneous loading	Non-quantitative tissue data	[30,39]	FLIM; ratiometric probes; light-sheet
Reversibility	Cumulative, not real-time	All triggers irreversible	Cannot track aldehyde flux	[23,24]	Equilibrium oxime designs; active research
Cell toxicity	Redox perturbation	Hydrazine reactivity	False positive; cell stress	[31,33]	Viability assays; lower-reactivity analogs
Water solubility	Aggregation	Hydrophobic fluorophore cores	Weak/variable cell signal	[27,30]	PEGylation; AIE scaffolds; zwitterionic

## Data Availability

No new data were created or analyzed in this study. Data sharing is not applicable to this article.

## References

[1] Brewer T.F., Chang C.J. (2015). An aza-Cope reactivity-based fluorescent probe for imaging formaldehyde in living cells. J. Am. Chem. Soc..

[2] Du Y., Zhang Y., Huang M., Wang S., Wang J., Liao K., Wu X., Zhou Q., Zhang X., Wu Y.-D. (2021). Systematic investigation of the aza-Cope reaction for fluorescence imaging of formaldehyde in vitro and in vivo. Chem. Sci..

[3] Tenney L., Pham V.N., Brewer T.F., Chang C.J. (2024). A mitochondrial-targeted activity-based sensing probe for ratiometric imaging of formaldehyde reveals key regulators of the mitochondrial one-carbon pool. Chem. Sci..

[4] Tenney L., Wen K.-K., Han S.-S., Vyas Y.M., Chang C.J. (2026). A nuclear-targeted activity-based sensing probe for ratiometric imaging of formaldehyde reveals endogenous epigenetic contributors to the nuclear formaldehyde pool. Chem. Sci..

[5] Tenney L., Pham V.N., Chang C.J. (2024). One carbon to rule them all: Formaldehyde is a one-carbon signal connecting one-carbon metabolism and epigenetic methylation. ACS Chem. Biol..

[6] Xu H., Zhang C., Zhang Y.Q., Suo S.-N., Wang Y.-W., Peng Y. (2022). A red-NIR fluorescent probe for rapid and visual detection of acrolein. Chem. Commun..

[7] Catalano A., Mariconda A., D’Amato A., Iacopetta D., Ceramella J., Marra M., Saturnino C., Sinicropi M.S., Longo P. (2024). Aldehydes: What we should know about them. Organics.

[8] Duan Q., Wang Y., Han J., Yu J., Jing J., Zhang R., Zhang X. (2024). Visualization of acrolein upregulation during ferroptosis by a ratiometric fluorescent probe. Anal. Chem..

[9] Chen J., Zeng L., Xia T., Li S., Yan T., Wu S., Qiu G., Liu Z. (2015). Toward a biomarker of oxidative stress: A fluorescent probe for exogenous and endogenous malondialdehyde in living cells. Anal. Chem..

[10] Peng M., Du Q., Yao X., Li C.-N., Tian Y., Peng Y., Wang Y.-W. (2023). A fluorescein-based fluorescent probe for fast detection of malondialdehyde and its imaging study. Org. Biomol. Chem..

[11] Zhang X., Li G., Li Y., Li N., Pan W., Tang B. (2025). A malondialdehyde-activated fluorescent probe reveals lysosomal dysfunction in atherosclerosis. Chem. Sci..

[12] Mukherjee K., Chio T.I., Gu H., Sackett D.L., Bane S.L., Sever S. (2021). A novel fluorogenic assay for the detection of nephrotoxin-induced oxidative stress in live cells and renal tissue. ACS Sens..

[13] Merck/MilliporeSigma Cancer Stem Cell Detection with AldeRed™ 588-A. Technical Article. https://www.sigmaaldrich.cn/CN/zh/technical-documents/technical-article/cell-culture-and-cell-culture-analysis/stem-cell-culture/aldered-aldh-cancer-stem-cell?srsltid=AfmBOorNonMHLT1YvU26LJV_t209M8IMtCbRC4yVrUcDJE6nVjlZoRyU.

[14] Dilek O. (2022). Current probes for imaging carbonylation in cellular systems and their relevance to progression of diseases. Technol. Cancer Res. Treat..

[15] Zhang W., Zhu H., Xie W., Du C., Fang X., Zhang R., Hu X., Lin Y. (2024). Highly sensitive analysis of fatty aldehydes in vegetable oils using a novel coumarin-based fluorescent probe by HPLC for quality control. Microchem. J..

[16] Minn I., Wang H., Mease R.C., Byun Y., Yang X., Wang J., Leach S.D., Pomper M.G. (2014). A red-shifted fluorescent substrate for aldehyde dehydrogenase. Nat. Commun..

[17] Wills R., Shirke R., Hrncir H., Talbott J.M., Sad K., Spangle J.M., Gracz A.D., Raj M. (2024). Tunable fluorescent probes for detecting aldehydes in living systems. Chem. Sci..

[18] Semwal K., Das A.K. (2025). Recent progress in fluorescent chemosensors for selective aldehyde detection. RSC Adv..

[19] Shortall K., Djeghader A., Magner E., Soulimane T. (2021). Insights into aldehyde dehydrogenase enzymes: A structural perspective. Front. Mol. Biosci..

[20] Erkan H., Telci D., Dilek O. (2020). Design of fluorescent probes for bioorthogonal labeling of carbonylation in live cells. Sci. Rep..

[21] Chen A.-L., Lin Z.-J., Chang H.-Y., Wang T.-S.A. (2025). Chemoselective stabilized triphenylphosphonium probes for capturing reactive carbonyl species and regenerating covalent inhibitors with acrylamide warheads in cellulo. J. Am. Chem. Soc..

[22] Marchitti S.A., Brocker C., Stagos D., Vasiliou V. (2008). Non-P450 aldehyde oxidizing enzymes: The aldehyde dehydrogenase superfamily. Expert Opin. Drug Metab. Toxicol..

[23] Dirksen A., Dawson P.E. (2008). Rapid oxime and hydrazone ligations with aromatic aldehydes for biomolecular labeling. Bioconjug. Chem..

[24] Kölmel D.K., Kool E.T. (2017). Oximes and hydrazones in bioconjugation: Mechanism and catalysis. Chem. Rev..

[25] Kirby A., Suchý M., Shuhendler A.J. (2024). MRI probes for in vivo aldehyde sensing. Anal. Sens..

[26] Weissleder R. (2001). A clearer vision for in vivo imaging. Nat. Biotechnol..

[27] Hong G., Antaris A.L., Dai H. (2017). Near-infrared fluorophores for biomedical imaging. Nat. Biomed. Eng..

[28] Lichtman J.W., Conchello J.-A. (2005). Fluorescence microscopy. Nat. Methods.

[29] Loudet A., Burgess K. (2007). BODIPY dyes and their derivatives: Syntheses and spectroscopic properties. Chem. Rev..

[30] Lavis L.D., Raines R.T. (2008). Bright ideas for chemical biology. ACS Chem. Biol..

[31] Chen H.-W., Li H., Song Q.-H. (2018). BODIPY-substituted hydrazine as a fluorescent probe for rapid and sensitive detection of formaldehyde in aqueous solutions and in live cells. ACS Omega.

[32] Ding N., Li Z., Hao Y., Zhang C. (2022). Design of a new hydrazine moiety-based near-infrared fluorescence probe for detection and imaging of endogenous formaldehyde in vivo. Anal. Chem..

[33] Tan X., Hua M., Wang S., Xu L., Zhang G., Gou X. (2024). Synthesis of an amine moiety-based fluorescent probe and the relationship between the amino substitution position and formaldehyde detection performance. ChemistrySelect.

[34] Chen H.H., Waghorn P.A., Wei L., Tapias L.F., SchühLe D.T., Rotile N.J., Jones C.M., Looby R.J., Zhao G., Elliott J.M. (2017). Molecular imaging of oxidized collagen quantifies pulmonary and hepatic fibrogenesis. JCI Insight.

[35] Li J.B., Wang Q.Q., Yuan L., Wu Y.-X., Hu X.-X., Zhang X.-B., Tan W. (2016). A two-photon fluorescent probe for bio-imaging of formaldehyde in living cells and tissues. Analyst.

[36] Xin F., Tian Y., Gao C., Guo B., Wu Y., Zhao J., Jing J., Zhang X. (2019). A two-photon fluorescent probe for basal formaldehyde imaging in zebrafish and visualization of mitochondrial damage induced by FA stress. Analyst.

[37] Cheng F., Qiang T., Wang B., Ren L., Hu W., James T.D. (2024). Observation of formaldehyde-induced ER stress by an ER-targeting two-photon probe. Sens. Actuators B Chem..

[38] Zheng J.J., Liu W.C., Lu F.N., Tang Y., Yuan Z.Q. (2022). Recent progress in fluorescent formaldehyde detection using small molecule probes. J. Anal. Test..

[39] Duan X., Zhang M., Zhang Y.-H. (2023). Organic fluorescent probes for live-cell super-resolution imaging. Front. Optoelectron..

[40] Udhayakumari D. (2025). Mechanistic innovations in fluorescent chemosensors for detecting toxic ions: PET, ICT, ESIPT, FRET and AIE approaches. J. Fluoresc..

[41] Wu L., Li Z., Wang K., Groleau R.R., Rong X., Liu X., Liu C., Lewis S.E., Zhu B., James T.D. (2025). Advances in organic small molecule-based fluorescent probes for precision detection of liver diseases: A perspective on emerging trends and challenges. J. Am. Chem. Soc..

[42] Fei K., Zhang J., Yuan J., Xiao P. (2022). Present application and perspectives of organoid imaging technology. Bioengineering.

[43] Jana A., Baruah M., Samanta A. (2022). Activity-based fluorescent probes for sensing and imaging of reactive carbonyl species (RCSs). Chem. Asian J..

[44] Stress C.J., Schmidt P.J., Gillingham D.G. (2016). Comparison of boron-assisted oxime and hydrazone formations leads to the discovery of a fluorogenic variant. Org. Biomol. Chem..

[45] Schmidt P., Stress C., Gillingham D. (2015). Boronic acids facilitate rapid oxime condensations at neutral pH. Chem. Sci..

[46] Han G.S., Domaille D.W. (2021). Tuning the exchange dynamics of boronic acid hydrazones and oximes with pH and redox control. Org. Biomol. Chem..

[47] Dilek O., Lei Z., Mukherjee K., Bane S. (2015). Rapid formation of a stable boron–nitrogen heterocycle in dilute, neutral aqueous solution for bioorthogonal coupling reactions. Chem. Commun..

[48] Miller E.W., Albers A.E., Pralle A., Isacoff E.Y., Chang C.J. (2005). Boronate-based fluorescent probes for imaging cellular hydrogen peroxide. J. Am. Chem. Soc..

[49] Guo L., Yang M., Dong B., Lewman S., Van Horn A., Jia S. (2024). Engineering central substitutions in heptamethine dyes for improved fluorophore performance. JACS Au.

